# Responses of rat and mouse primary microglia to pro- and anti-inflammatory stimuli: molecular profiles, K^+^ channels and migration

**DOI:** 10.1186/s12974-017-0941-3

**Published:** 2017-08-22

**Authors:** Doris Lam, Starlee Lively, Lyanne C. Schlichter

**Affiliations:** 1 Genes and Development Division, Krembil Research Institute, University Health Network, Krembil Discovery Tower, Room 7KD417, 60 Leonard Avenue, Toronto, ON M5T 2S8 Canada; 2 Department of Physiology, University of Toronto, Toronto, ON Canada

**Keywords:** Microglia molecular polarization, M1, M2a, M2c activation, K^+^ channels, Kv1.3 channel, Kir2.1 channel, Microglial migration

## Abstract

**Background:**

Acute CNS damage is commonly studied using rat and mouse models, but increasingly, molecular analysis is finding species differences that might affect the ability to translate findings to humans. Microglia can undergo complex molecular and functional changes, often studied by in vitro responses to discrete activating stimuli. There is considerable evidence that pro-inflammatory (M1) activation can exacerbate tissue damage, while anti-inflammatory (M2) states help resolve inflammation and promote tissue repair. However, in assessing potential therapeutic targets for controlling inflammation, it is crucial to determine whether rat and mouse microglia respond the same.

**Methods:**

Primary microglia from Sprague-Dawley rats and C57BL/6 mice were cultured, then stimulated with interferon-γ + tumor necrosis factor-α (I + T; M1 activation), interleukin (IL)-4 (M2a, alternative activation), or IL-10 (M2c, acquired deactivation). To profile their activation responses, NanoString was used to monitor messenger RNA (mRNA) expression of numerous pro- and anti-inflammatory mediators, microglial markers, immunomodulators, and other molecules. Western analysis was used to measure selected proteins. Two potential targets for controlling inflammation—inward- and outward-rectifier K^+^ channels (Kir2.1, Kv1.3)—were examined (mRNA, currents) and specific channel blockers were applied to determine their contributions to microglial migration in the different activation states.

**Results:**

Pro-inflammatory molecules increased after I + T treatment but there were several qualitative and quantitative differences between the species (e.g., iNOS and nitric oxide, COX-2). Several molecules commonly associated with an M2a state differed between species or they were induced in additional activation states (e.g., CD206, ARG1). Resting levels and/or responses of several microglial markers (Iba1, CD11b, CD68) differed with the activation state, species, or both. Transcripts for several Kir2 and Kv1 family members were detected in both species. However, the current amplitudes (mainly Kir2.1 and Kv1.3) depended on activation state and species. Treatment-induced changes in morphology and migratory capacity were similar between the species (migration reduced by I + T, increased by IL-4 or IL-10). In both species, Kir2.1 block reduced migration and Kv1.3 block increased it, regardless of activation state; thus, these channels might affect microglial migration to damage sites.

**Conclusions:**

Caution is recommended in generalizing molecular and functional responses of microglia to activating stimuli between species.

**Electronic supplementary material:**

The online version of this article (doi:10.1186/s12974-017-0941-3) contains supplementary material, which is available to authorized users.

## Background

Rats have been used for many years to model CNS damage and disease because they have many physiological similarities to humans and can learn a wide variety of tasks, which makes them useful for behavioral studies [1]. More recently, mice have been increasingly favored because of the ease of genetic manipulation [2, 3], although transgenic technology in rats is now advancing [4]. Immune responses of mice and humans are increasingly being compared [5–7], and it is crucial to determine the similarities and differences between the commonly used rodent species. However, surprisingly few studies of microglia have compared their responses in both rodent species [8, 9], and this knowledge gap could affect the ability to translate experimental findings to human treatments.

When the CNS is injured, brain cells release “damage-associated molecular pattern” molecules (or “alarmins”) and other soluble mediators, including cytokines, high-mobility group box 1, purine metabolites, and nucleic acids. In response, microglia “activate”, and this is accompanied by dramatic morphological and molecular changes [10, 11]. There is increasing interest in assessing inflammatory responses to CNS injury, and a recent view is that microglial activation evolves as a continuum over time [10–12]. It is well established that microglia can assume multiple activation states, and there has been a focus on identifying markers to distinguish between pro- and anti-inflammatory states. Changes in activation states are also expected to affect functional outcomes, including the capacity of microglia to produce immune mediators, migrate, proliferate, and phagocytose dead cells and debris. To elucidate responses to stimuli that can skew microglia toward a particular activation state, molecular profiles and cell functions are normally assessed in vitro.

The terminology for microglial activation is evolving [10, 13–16]. For clarity, we will use the following. “Classical” activation (M1), which is a pro-inflammatory phenotype thought to exacerbate tissue damage, is usually induced in vitro by bacterial lipopolysaccharide (LPS) with or without IFN-γ. However, to better reflect stimuli that are present after acute CNS, including stroke, we now use a combination of IFN-γ and TNF-α to induce a pro-inflammatory state [17], which we denote as M(I + T). Several anti-inflammatory (M2) states have been implicated in tissue repair, matrix deposition, and resolution of pro-inflammatory states. Of these, “alternative activation” (M2a; induced by IL-4 and/or IL-13) and “acquired deactivation” (M2c; induced by IL-10, TGF-β1 or glucocorticoids) have received the most attention. Here, we assessed an M(IL-4) state and an M(IL-10) state. Microglial activation states are usually identified by altered expression of marker molecules, but less is known about functional correlates. Recently, we reported that several functions of rat microglia are activation state dependent. Migration was drastically reduced in a M(LPS) state but increased in M(IL-4) and M(IL-10) states [18–20], and myelin phagocytosis was increased in M(I + T) and M(IL-10) states but was unaffected in an M(IL-4) state [17].

In attempting to identify therapeutic targets to modulate microglial activation, numerous studies are addressing the expression and contributions of several K^+^ channels. Following acute CNS injury, rodent microglia in situ express inward-rectifier and outward-rectifier K^+^ currents [21–23] but their prevalence is controversial. In vitro studies have implicated Kir2.1 [18, 24–26], and Kv1.3 channels [26–28] in several microglia functions. However, there is some evidence that expression of Kir and Kv currents can change with microglial activation and this is expected to affect channel contributions to cell functions. For instance, in rat microglia, Kir2.1 activity is required for migration under unstimulated M(IL-4) and M(IL-10) states [18], whereas, in M(LPS) cells, Kv1.3 expression increased and contributed to neurotoxicity [29]. Published results hint at differences in Kv1.3 and Kir2.1 currents between rat and mouse microglia.

The present study directly compares numerous molecular responses, as well as some functional outcomes in primary microglia from rat and mouse. We compared responses to the pro-inflammatory stimulus, I + T, and the anti-inflammatory stimuli, IL-4 and IL-10. First, we profiled a wide variety of pro- and anti-inflammatory mediators, receptors, activation markers, and immune modulators. Then, we compared Kir2.1 and Kv1.3 expression and channel activity (currents) and examined their involvement in microglial migration. The results show similarities and differences between these rodent species that should be considered when characterizing microglial activation in vitro and in vivo.

## Methods

### Microglia isolation, culturing, and activation

All procedures on animals were approved by the University Health Network Animal Care Committee (Animal Use Protocols 914 and 1573) and adhered to the Canadian Council on Animal Care guidelines for humane animal use. Pure neonatal microglia cultures were prepared from Sprague-Dawley rat pups (P1–P2) and C57BL/6 mouse pups (P0–P2). We selected this outbred rat strain and inbred mouse strain because they are both widely used in biomedical research, and specifically because C57BL/6 mice are the primary strain used in transgenic studies. Animals were purchased from Charles River (St-Constant, PQ, Canada). As we recently described for rat microglia [18–20, 30, 31], brain tissue (excluding the cerebellum and meninges) was mashed, strained, and centrifuged (300×*g*, 10 min) in cold Minimal Essential Medium (MEM; Invitrogen, Carlsbad, CA). The pellet was re-suspended in MEM and seeded in 75-cm^2^ flasks containing 20 mL of MEM supplemented with 10% fetal bovine serum (FBS; Wisent St-Bruno, PQ) and 0.05 mg/mL gentamycin (Invitrogen). Cells were incubated at 37 °C with 5% CO_2_, and after 48 h, the medium was changed to remove cellular debris and non-adherent cells. After 5–6 days (rat) or 10–14 days (mouse), microglia were harvested by shaking the flasks (5 h, 65 rpm) on an orbital shaker in the incubator (37 °C, 5% CO_2_). The supernatant containing microglia was collected, centrifuged, and re-suspended in fresh MEM (2% FBS, 0.05 mg/mL gentamycin). Microglia were seeded onto UV-irradiated 15-mm glass coverslips (Fisher Scientific, Ottawa, ON) at different densities based on the experiment, as detailed below. For mouse microglia, it is difficult to obtain the large numbers of cells needed to perform the multiple treatments, functional assays, and Western blot analyses. Rather than using a cell line (e.g., BV2, which does not necessarily respond the same as primary microglia [32, 33], we grew the cells longer to expand the population. Importantly, we confirmed that expression of numerous genes (see “Results” below) shows that their initial “resting” state was similar to rat microglia, and that many activation responses were similar between the species. Thus, where specific differences were seen between the species, they are unlikely to reflect culturing times.

After seeding, microglia were allowed to settle for 2–3 days (37 °C, 5% CO_2_), and then were left unstimulated (control; CTL) or stimulated with 20 ng/mL IFN-γ plus 50 ng/mL TNF-α to induce a pro-inflammatory state [M(I + T)], or with 20 ng/mL IL-4 [M(IL-4)] or 20 ng/mL IL-10 [M(IL-10)] to induce anti-inflammatory states. The recombinant cytokines (R & D Systems Inc., Minneapolis, MN) were specific to the rodent species; e.g., mIL-4 was used on mouse microglia. For messenger RNA (mRNA), protein, and functional analyses (nitric oxide production, migration), the cells were stimulated for 24 h, which was chosen to facilitate comparisons with our previous studies of rat microglia [17–20, 30, 34, 35], which show that many genes respond at 24 h. For electrophysiological analysis, cells were used 30 h after stimulation to provide additional time for channel trafficking and post-translational modifications.

### Multiplexed gene expression analysis (NanoString nCounter)

Microglia were seeded at 5 × 10^5^ cells/coverslip in a 12-well culture plate, allowed to settle for 1–2 days (37 °C, 5% CO_2_), and then stimulated with cytokines for 24 h. Total RNA was extracted using TRIzol reagent (Invitrogen) and purified using an RNeasy Mini Kit (QIAGEN, Mississauga, ON, Canada), as previously described [17–19, 30]. Samples were stored at −80 °C and used for NanoString and real-time qRT-PCR assays.

For NanoString analysis, extracted RNA (200 ng per sample) was sent to the Princess Margaret Genomics Centre (https://www.pmgenomics.ca/pmgenomics/; Toronto, Canada), where sample purity was assessed (using Nanodrop 1000), and the assay was conducted (hybridization, detection, scanning) using samples from each rodent species. We analyzed the data using nSolver Analysis Software (ver3.0). The methods were similar to our recent studies of primary rat microglia [17, 20, 30]; nevertheless, it is useful to more fully describe the controls and normalization procedures. NanoString is a medium-throughput method that can analyze many genes in a single sample with comparable sensitivity and accuracy to quantitative real-time RT-PCR (qRT-PCR) [36]. Moreover, by eliminating the need for amplification, it reports mRNA counts in a given sample, is more sensitive and accurate than microarrays [36], and in fact, is sometimes used to validate microarray data [37, 38]. Many NanoString studies have not directly compared the results with qRT-PCR (e.g., [39–44]) but a recent study directly compared qRT-PCR with NanoString and the ABI OpenArray System (another medium-throughput platform) [45]. While overall trends in mRNA expression were similar using all three platforms, NanoString and OpenArray results were better correlated.

Separate plates had to be used for each species, and different probe sets were designed and synthesized by NanoString nCounter technologies (rat, Table 1; mouse, Table 2. Note that gene names sometimes differ slightly between species.) Each transcript of interest was recognized by a capture probe and a reporter probe, each containing 30–50 bases complementary to the target mRNA. To minimize assay variability, the code sets also included negative and positive control reporter probes that were developed by the External RNA Control Consortium (ERCC). The eight negative-control reporter probes representing foreign sequences (not homologous to any organism) are not expected to detect the foreign transcripts in the samples. These background levels were calculated for each sample (geometric mean counts) and subtracted from the raw counts for each gene. Six positive control reporter probes (ERCC-selected mRNA targets) were pre-mixed with (Spike-Ins) the code set at a concentration range (0.125–128 fM), a range corresponding to the expression levels of most mRNA of interest, to control for overall efficiency of probe hybridization and determine the detection range for transcripts of interest in each assay. A scaling factor was determined, as follows. For each positive control probe, the geometric mean was calculated from counts obtained from each microglia sample, and the geometric means of all six positive controls were then averaged. This mean was divided by the geometric mean of the positive controls within a given microglia sample to obtain a sample-specific positive control scaling factor. A scaling factor outside the range of 0.3 to 3 indicates suboptimal hybridization. In our samples, the scaling factor always fell within the optimal range and was thus applied to all counts in the sample. Next, a reference gene scaling factor was calculated in the same manner using two housekeeping genes (*Hprt1*, *Gusb*), and used to adjust the counts for each sample (unstimulated or stimulated microglia). Sometimes, expression of a gene was very low (<20 mRNA counts/200 ng sample), which approaches the detection limit and should be treated with caution.

**Table 1:** Rat target sequences used to create Custom CodeSet for nCounter Assay

Gene	Accession #	Target sequence
*Aif1*	NM_017196.2	ATCGATATTATGTCCTTGAAGCGAATGCTGGAGAAACTTGGGGTTCCCAAGACCCATCTAGAGCTGAAGAAATTAATTAGAGAGGTGTCCAGTGGCTCCG
*Arg1*	NM_017134.2	ACGGGAAGGTAATCATAAGCCAGAGACTGACTACCTTAAACCACCGAAATAAATGTGAATACATCGCATAAAAGTCATCTGGGGCATCACAGCAAACCGA
*Casp1*	NM_012762.2	AGATTCTAAGGGAGGACATCCTTTCTCCTCAGAAACAAAAGAAAAACTGAACAAAGAAGGTGGCGCATTTCCTGGACCGAGTGGTTCCCTCAAGTTTTGC
*Ccl22*	NM_057203.1	TACATCCGTCACCCTCTGCCACCACGTTTCGTGAAGGAGTTCTACTGGACCTCAAAGTCCTGCCGCAAGCCTGGCGTCGTTTTGATAACCATCAAGAACC
*Cd163*	NM_001107887.1	CCTCTGTAATTTGCTCAGGAAACCAATCGCATACACTGTTGCCATGTAGTTCATCATCTTCGGTCCAAACAACAAGTTCTACCATTGCAAAGGACAGTGA
*Cd68*	NM_001031638.1	CTCTCATTCCCTTACGGACAGCTTACCTTTGGATTCAAACAGGACCGACATCAGAGCCACAGTACAGTCTACCTTAACTACATGGCAGTGGAATACAATG
*Cx3cr1*	NM_133534.1	ATGTGCAAGCTCACGACTGCTTTCTTCTTCATTGGCTTCTTTGGGGGCATATTCTTCATCACCGTCATCAGCATCGACCGGTACCTCGCCATCGTCCTGG
*Cybb*	NM_023965.1	CAGTACCAAAGTTTGCCGGAAACCCTCCTATGACTTGGAAATGGATCGTGGGTCCCATGTTCCTGTATCTGTGTGAGAGGCTGGTGCGGTTTTGGCGATC
*Fcgr1a*	NM_001100836.1	TGATGGATCATACTGGTGCGAGGTAGCCACGGAGGACGGCCGTGTCCTTAAGCGCAGCACCAAGTTGGAGCTATTTGGTCCCCAGTCATCAGATCCTGTC
*Fcgr2b*	NM_175756.1	CTGGTCCAAGGAATGCTGTAGATATGAAAGAAAACATCTAGAGTCCCTTCTGTGAGTCCTGAAACCAACAGACACTACGATATTGGTTCCCAATGGTTGA
*Fcgr3a*	NM_207603.1	GACTCTTGTTTGCAATAGACACAGTGCTGTATTTCTCGGTGCAGAGGAGTCTTCAAAGTTCCGTGGCAGTCTATGAGGAACCCAAACTTCACTGGAGCAA
*Gusb*	NM_017015.2	TCATTTGATCCTGGATGAGAAACGAAAAGAATATGTCATCGGAGAGCTCATCTGGAATTTTGCTGACTTCATGACGAACCAGTCACCACTGAGAGTAACA
*Hprt1*	NM_012583.2	AGCTTCCTCCTCAGACCGCTTTTCCCGCGAGCCGACCGGTTCTGTCATGTCGACCCTCAGTCCCAGCGTCGTGATTAGTGATGATGAACCAGGTTATGAC
*Ifng*	NM_138880.2	AAGGACGGTAACACGAAAATACTTGAGAGCCAGATTATCTCTTTCTACCTCAGACTCTTTGAAGTCTTGAAAGACAACCAGGCCATCAGCAACAACATAA
*Ifngr1*	NM_053783.1	CCTGTTACACATTCGACTACACTGTGTTTGTGAAACATTACAGGAGTGGGGAGATCCTACATACAGAACATAGCGTCCTAAAAGAAGATTGTAGCGAAAC
*Ifngr2*	NM_001108313.1	TTTCTTAAGTTACACTTAGTAAAGCAGATGAGTCCGCAGGAGACTTCAGCAAGAAAGAAGTTCCTACCGTCTCATCCCTTAGTTCTTCAAAGCCAAAGGA
*Il10*	NM_012854.2	ACAACATACTGCTGACAGATTCCTTACTGCAGGACTTTAAGGGTTACTTGGGTTGCCAAGCCTTGTCAGAAATGATCAAGTTTTACCTGGTAGAAGTGAT
*Il10ra*	NM_057193.2	TGTTTACATGTCACGACGGAGCATTATTTCACCGTGACCAACCTCAGCATTTTCTTCTTATCCATCCTGATACTCTGTGGAGCCCTGGTCTGCCTGGTTC
*Il10rb*	NM_001107111.1	CCTCCCTGGATCGTGGCCATCATCCTTATAGCCTCCGTCTTGATAGTCTTCCTCTTCCTACTGGGCTGCTTCAGCATGGTGTGGTTCATTTACAAGAAGA
*Il13ra1*	NM_145789.2	TAACGAATTTGAGTGTCTCTGTCGAAAATCTCTGCACAATAGTGTGGACATGGAGTCCTCCTGAGGGAGCCAGTCCAAATTGCAGTCTCAGATATTTTAG
*Il1b*	NM_031512.1	TGCACTGCAGGCTTCGAGATGAACAACAAAAATGCCTCGTGCTGTCTGACCCATGTGAGCTGAAAGCTCTCCACCTCAATGGACAGAACATAAGCCAACA
*Il1r1*	NM_013123.3	CTCATATTCTGGAGACTGCACACGTACGGTTAGTATACCCAGTTCCTGACTTCAAGAATTACCTCATCGGGGGCTTTGCCATCTTCACAGCTACAGCCGT
*Il1rn*	NM_022194.2	TCATTGCTGGGTACTTACAAGGACCAAATACCAAACTAGAAGAAAAGATAGACATGGTGCCTATTGACTTTCGGAATGTGTTCTTGGGCATCCACGGGGG
*Il4*	NM_201270.1	TGCTGTCACCCTGTTCTGCTTTCTCATATGTACCGGGAACGGTATCCACGGATGTAACGACAGCCCTCTGAGAGAGATCATCAACACTTTGAACCAGGTC
*IL4r*	NM_133380.2	GGGTGTCAGCATCTCCTGCATCTGCATCCTATTGTTTTGCCTGACCTGTTACTTCAGCATTATCAAGATTAAGAAGATATGGTGGGACCAGATTCCCACT
*Il6*	NM_012589.1	GGAACAGCTATGAAGTTTCTCTCCGCAAGAGACTTCCAGCCAGTTGCCTTCTTGGGACTGATGTTGTTGACAGCCACTGCCTTCCCTACTTCACAAGTCC
*Itgam*	NM_012711.1	CATCCCTTCCTTCAACAGTAAAGAAATATTCAACGTCACCCTCCAGGGCAATCTGCTATTTGACTGGTACATCGAGACTTCTCATGACCACCTCCTGCTT
*Kcna2*	NM_012970.3	GCCGGCCAGGATCATAGCCATTGTATCTGTGATGGTCATTCTGATCTCGATCGTCAGCTTCTGTCTGGAAACCTTGCCCATCTTCCGGGATGAGAACGAG
*Kcna3*	NM_019270.3	GCCACCTTCTCCAGAAATATCATGAACCTGATAGACATTGTAGCCATCATCCCTTATTTTATTACTCTGGGCACTGAGCTGGCTGAGCGACAGGGTAATG
*Kcna5*	NM_012972.1	ATCAGAAGGGGTAGCTGTCCTCTAGAAAAGTGTCACCTCAAGGCCAAGAGCAACGTGGACTTGCGGAGGTCCCTGTATGCCCTCTGTCTGGACACTAGCC
*Kcnj2*	NM_017296.1	GTTCTTTGGCTGTGTGTTTTGGTTGATAGCTCTGCTCCACGGGGATCTGGATGCTTCTAAAGAGAGCAAAGCGTGTGTGTCTGAGGTCAACAGCTTCACG
*Mrc1*	NM_001106123.1	CTTTGGAATCAAGGGCACAGAGCTATATTTTAACTATGGCAACAGGCAAGAAAAGAATATCAAGCTTTACAAAGGTTCCGGTTTGTGGAGCAGATGGAAG
*Msr1*	NM_001191939.1	CACGTTCCATGACAGCATCCCTTCCTCACAACACTATAAATGGCTCCTCCGTTCAGGAGAAACTGAAGTCCTTCAAAGTTGCCCTCGTCGCTCTCTACCT
*Myc*	NM_012603.2	ACCGAGGAAAACGACAAGAGGCGGACACACAACGTCTTGGAACGTCAGAGGAGAAACGAGCTGAAGCGTAGCTTTTTTGCCCTGCGCGACCAGATCCCTG
*Ncf1*	NM_053734.2	TCCATTCCCAGCATCCCATAATTGGGCTTGTCCGTGTTCCAACATCTGGGCGGAATTTCACAGCCAAAGGTCAAGAGGACTGCTGTTACGTTCAAGGTCG
*Nfkbia*	NM_001105720.2	TATTGTGCTTTTGGTTGAACCGCCATAGACTGTAGCTGACCCCAGTGTGCCCTCTCACGTAAGAACCAGGTGTTCAGTGGTATGTGCTTAAGTCATCCCC
*Nos2*	NM_012611.2	ACGGGACACAGTGTCGCTGGTTTGAAACTTCTCAGCCACCTTGGTGAGGGGACTGGACTTTTAGAGACGCTTCTGAGGTTCCTCAGGCTTGGGTCTTGTT
*Nox1*	NM_053683.1	CCGAGAAAGAAGATTCTTGGCTAAATCCCATCCAGTCTCCAAACGTGACAGTGATGTATGCAGCATTTACCAGTATTGCTGGCCTTACTGGAGTGGTCGC
*Nox4*	NM_053524.1	TGTTGGACAAAAGCAAGACTCTACATATCACCTGTGGCATAACTATTTGTATTTTCTCAGGTGTGCATGTAGCTGCCCACTTGGTGAACGCCCTGAACTT
*Nr3c1*	NM_012576.2	AGCTTTCCTTGAAGCGTATAAAGAGCCATGCTCCTTTAGTATGTGGGGAAGAAGAGAGCTGTCATAGTTTTGAGTACAGTGAGAAGATGCGGTACTGTCT
*P2rx7*	NM_019256.1	ACTTTAAGAGGTCACATTAACCAGACTAGAAGCCATCGCATCTAACCGCATACCAGACACAGTCTGACGCCTCATTGCTATGCTATGGTTCTAAGTGACT
*P2ry12*	NM_022800.1	TGATAACCATTGACCGATACCTGAAGACCACCAGACCATTTAAAACTTCCAGCCCCAGCAATCTTTTGGGTGCGAAGATTCTTTCTGTTGCCATCTGGGC
*P2ry2*	NM_017255.1	GAGCTCTTTAGCCATTTTGTGGCTTACAGCTCTGTCATGCTGGGTCTGCTTTTTGCTGTGCCCTTTTCCATCATCCTGGTCTGTTACGTGCTCATGGCCC
*Pparg*	NM_013124.1	TTTATAGCTGTCATTATTCTCAGTGGAGACCGCCCAGGCTTGCTGAACGTGAAGCCCATCGAGGACATCCAAGACAACCTGCTGCAGGCCCTGGAACTCC
*Ptgs2*	NM_017232.3	TTCGGAGGAGAAGTGGGTTTTAGGATCATCAACACTGCCTCAATTCAGTCTCTCATCTGCAATAATGTGAAAGGGTGTCCCTTTGCCTCTTTCAATGTGC
*Ptk2b*	NM_017318.2	GCAGTGATCATGAAGAATCTTGACCACCCTCACATCGTCAAGCTGATTGGCATCATTGAAGAGGAACCCACATGGATCGTCATGGAACTGTATCCTTATG
*Ptpn6*	NM_053908.1	GCAGAGTCACTGCTGCAGGCCAAGGGCGAGCCCTGGACATTTCTTGTGCGTGAGAGTCTCAGCCAACCTGGTGATTTTGTGCTCTCTGTGCTCAATGACC
*Retnla*	NM_053333.1	AGGAACTTCTAGCCCATCAAGATAACTATCCCTCTGCTGTAAGGAAGACCCTCTCATGCACTAATGTCAAGTCTATGAGCAAATGGGCCTCCTGCCCTGC
*Socs1*	NM_145879.1	CGGCCGCTGCAGGAGCTGTGTCGCCAGCGCATCGTGGCCGCCGTGGGTCGCGAGAACCTGGCACGCATCCCTCTTAACCCGGTACTCCGTGACTACCTGA
*Socs3*	NM_053565.1	GGAAGACTGTCAACGGTCACCTGGACTCCTATGAGAAAGTGACCCAGCTGCCTGGACCCATTCGGGAGTTCCTGGACCAGTATGATGCTCCACTTTAAAG
*Tgfb1*	NM_021578.2	CGCCTGCAGAGATTCAAGTCAACTGTGGAGCAACACGTAGAACTCTACCAGAAATATAGCAACAATTCCTGGCGTTACCTTGGTAACCGGCTGCTGACCC
*Tgfbr1*	NM_012775.2	GTCTGCATTGCACTTATGCTGATGGTCTATATCTGCCATAACCGCACTGTCATTCACCACCGCGTACCAAATGAAGAGGATCCCTCACTAGATCGCCCTT
*Tgfbr2*	NM_031132.3	CCAGCAGTCCTGACCTGTTGCTGGTCATTATCCAAGTGACGGGCGTCAGCCTCCTGCCTCCGCTGGGGATTGCCATAGCTGTCATTGCCATCTTCTACTG
*Tlr2*	NM_198769.2	TTTACAAACCCTTAGGGTAGGAAATGTTGACACTTTCAGTGAGATAAGGAGAATAGATTTTGCTGGGCTGACCTCTCTCAACGAACTTGAAATTCAGGTA
*Tlr4*	NM_019178.1	GTCAGTGTGCTTGTGGTAGCCACTGTAGCATTTCTGATATACCACTTCTATTTTCACCTGATACTTATTGCTGGCTGTAAAAAGTACAGCAGAGGAGAAA
*Tnf*	NM_012675.2	GGTGATCGGTCCCAACAAGGAGGAGAAGTTCCCAAATGGGCTCCCTCTCATCAGTTCCATGGCCCAGACCCTCACACTCAGATCATCTTCTCAAAACTCG
*Tnfrsf1a*	NM_013091.1	TATTCTTTATCTGCATCAGTCTACTGTGCCGATATCCCCAGTGGAGGCCCAGGGTCTACTCCATCATTTGTAGGGATTCAGCTCCTGTCAAAGAGGTGGA
*Tnfrsf1b*	NM_130426.4	AGGAGTTCAGATTCTTCCCATGGCAGCCACGGGACCCATGTCAACGTCACCTGCATCGTGAACGTCTGTAGCAGCTCTGACCACAGCTCTCAGTGTTCTT
*Trem2*	NM_001106884.1	TCCGGCTGGCTGAGGAAGGGTGCCATGGAACCTCTCCACGTGTTTGTCCTGTTGCTGGTCACAGAGCTGTCCCAAGCCCTCAACACCACAGTGCTGCAGG
*Tspo*	NM_012515.1	GCTGCCCGCTTGCTGTATCCTTACCTGGCCTGGCTGGCCTTTGCCACCATGCTCAACTACTATGTATGGCGTGATAACTCTGGTCGGCGAGGGGGCTCCC

**Table 2:** Mouse target sequences used to create Custom CodeSet for nCounter Assay

Gene	Accession #	Target sequence
*Aif1*	NM_019467.2	CTGGAGCAGCCTGCAGACTTCATCCTCTCTCTTCCATCCCGGGGAAAGTCAGCCAGTCCTCCTCAGCTGCCTGTCTTAACCTGCATCATGAAGCCTGAGG
*Arg1*	NM_007482.3	GTACATTGGCTTGCGAGACGTAGACCCTGGGGAACACTATATAATAAAAACTCTGGGAATTAAGTATTTCTCCATGACTGAAGTAGACAAGCTGGGGATT
*Casp1*	NM_009807.2	GACAATAAATGGATTGTTGGATGAACTTTTAGAGAAGAGAGTGCTGAATCAGGAAGAAATGGATAAAATAAAACTTGCAAACATTACTGCTATGGACAAG
*Ccl22*	NM_009137.2	CCAAGAATCAACTTCCACCCCTCTTCAACCACATGCTAGGGTCTTTTACTTTCTCTGCCCCACACCTTTGACTCCTTGCCTGTGTAGCTGATAGTCGAAG
*Cd163*	NM_053094.2	TCACGGCACTCTTGGTTTGTGGAGCCATTCTATTGGTCCTCCTCATTGTCTTCCTCCTGTGGACTCTGAAGCGACGACAGATTCAGCGACTTACAGTTTC
*Cd68*	NM_009853.1	GCTCCCTGTGTGTCTGATCTTGCTAGGACCGCTTATAGCCCAAGGAACAGAGGAAGACTGTCCTCACAAAAAGGCCGTTACTCTCCTGCCATCCTTCACG
*Cx3cr1*	NM_009987.3	TATGCTTTGGTGTTGGTCTGTATTTCCCGCTGTCTCGGGTCACATGGTTAAGCGTGCCTAGAGTGTGTCTATCCCACTTGTAATTCTGTCAATAAACATT
*Cybb* )	NM_007807.2	ACAGAAGACTCTGTATGGACGGCCCAACTGGGATAACGAGTTCAAGACCATTGCAAGTGAACACCCTAACACCACAATAGGCGTTTTCCTGTGTGGCCCT
*Fcgr1a*	NM_010186.5	GAGACAGTTCCACACAATGGTTTATCAACGGAACAGCCGTTCAGATCTCCACGCCTAGTTATAGCATCCCAGAGGCCAGTTTTCAGGACAGTGGCGAATA
*Fcgr2b*	NM_001077189.1	TTGGTTCCCAATGGTTGACTGTACTAATGACTCCCATAACTTACAGCTTCCCAACTCAAGACTCTTCTGCTATCGATCCACACTGCCACTAAAATTAATC
*Fcgr3a*	NM_010188.5	TCTGACCTCCACCATCCACCATGGCAGGTGCACACAATAAATTAAAATGTCATGTATATTTTTAAACAAGAGACAGGGGCAGGCTAAGGGTTGATGGCAT
*Gusb*	NM_010368.1	AATACGTGGTCGGAGAGCTCATCTGGAATTTCGCCGACTTCATGACGAACCAGTCACCGCTGAGAGTAATCGGAAACAAGAAGGGGATCTTCACTCGCCA
*Hprt*	NM_013556.2	TGCTGAGGCGGCGAGGGAGAGCGTTGGGCTTACCTCACTGCTTTCCGGAGCGGTAGCACCTCCTCCGCCGGCTTCCTCCTCAGACCGCTTTTTGCCGCGA
*Ifng*	NM_008337.1	CTAGCTCTGAGACAATGAACGCTACACACTGCATCTTGGCTTTGCAGCTCTTCCTCATGGCTGTTTCTGGCTGTTACTGCCACGGCACAGTCATTGAAAG
*Ifngr1*	NM_010511.2	AAGCATAATGTTACCTAAGTCCTTGCTCTCTGTGGTAAAAAGTGCCACGTTAGAGACAAAACCTGAATCGAAGTATTCACTTGTCACACCGCACCAGCCA
*Ifngr2*	NM_008338.3	CATCCTGATTCCGTTGGGCATCTTCGCATTGCTGCTCGGCCTGACGGGCGCCTGCTTCACCCTGTTCCTCAAATACCAAAGCCGAGTGAAGTACTGGTTT
*Il10*	NM_010548.1	GGGCCCTTTGCTATGGTGTCCTTTCAATTGCTCTCATCCCTGAGTTCAGAGCTCCTAAGAGAGTTGTGAAGAAACTCATGGGTCTTGGGAAGAGAAACCA
*Il10ra*	NM_008348.2	TGTTGTCGCGTTTGCTCCCATTCCTCGTCACGATCTCCAGCCTGAGCCTAGAATTCATTGCATACGGGACAGAACTGCCAAGCCCTTCCTATGTGTGGTT
*Il10rb*	NM_008349.5	CTTTACACCTGCGTTTCTCAGCCCCACAAATTGAGAATGAGCCTGAGACGTGGACCTTGAAGAACATTTATGACTCATGGGCTTACAGAGTGCAATACTG
*Il13ra1*	NM_133990.4	CTCAAACCGACCGACATAATATTTTAGAGGTTGAAGAGGACAAATGCCAGAATTCCGAATCTGATAGAAACATGGAGGGTACAAGTTGTTTCCAACTCCC
*Il1b*	NM_008361.3	GTTGATTCAAGGGGACATTAGGCAGCACTCTCTAGAACAGAACCTAGCTGTCAACGTGTGGGGGATGAATTGGTCATAGCCCGCACTGAGGTCTTTCATT
*Il1r1*	NM_001123382.1	CTTCTTCGGAGTAAAAGATAAACTGTTGGTGAGGAATGTGGCTGAAGAGCACAGAGGGGACTATATATGCCGTATGTCCTATACGTTCCGGGGGAAGCAA
*Il1rn*	NM_031167.5	CAACCAGCTCATTGCTGGGTACTTACAAGGACCAAATATCAAACTAGAAGAAAAGATAGACATGGTGCCTATTGACCTTCATAGTGTGTTCTTGGGCATC
*Il4*	NM_021283.1	TGCTTGAAGAAGAACTCTAGTGTTCTCATGGAGCTGCAGAGACTCTTTCGGGCTTTTCGATGCCTGGATTCATCGATAAGCTGCACCATGAATGAGTCCA
*IL4ra*	NM_001008700.3	CCCACAGCAGTGCTGACGTTCCTAAGTCCTGGGCTTTCCTAGCTGATGTTGTCCTACCTACTCAGTCCCATTTTGTCCACCGAATAGACCTGTCACTCAA
*Il6*	NM_031168.1	CTCTCTGCAAGAGACTTCCATCCAGTTGCCTTCTTGGGACTGATGCTGGTGACAACCACGGCCTTCCCTACTTCACAAGTCCGGAGAGGAGACTTCACAG
*Itgam*	NM_001082960.1	ATCCCTGTTCAGATCAACAATGTGACCGTATGGGATCATCCCCAGGTCATCTTCTCCCAGAACCTCTCAAGTGCCTGTCACACTGAGCAGAAATCCCCCC
*Kcna2*	NM_008417.4	GTTAACTGATGTCTGATTGAAGCCTACTAATGTACTCACAGCTCAACAGGACTGATGCAGATGTTGCATAATAGCCTGCATTGTAGTCAGTGTTCTACAG
*Kcna3*	NM_008418.2	CTGTTGGTTATGGTGATATGCACCCAGTGACCATAGGAGGCAAGATTGTGGGCTCTCTTTGTGCCATCGCAGGTGTCTTGACCATTGCATTGCCAGTTCC
*Kcna5*	NM_145983.2	AAAAAGTATCGCATTCCATGACGCAGGAGCCGTTGAAGTGGTGAGCATTCACTGTAAGATGGATGTATTCATAGCCAGTTTTCTATACCCAGCAGAGGGA
*Kcnj2*	NM_008425.4	CTTAAGGCGAGAATCGGAGATATGACTGGCTGATTCCGTCTTTGGAATACTTACTTTGCTACACAGCCTGACGTTGGTCAGAGGTCCGAGACAGTTATAC
*Mrc1*	NM_008625.1	GTTCCGAAATGTTGAAGGGAAGTGGCTTTGGTTGAACGACAATCCTGTCTCCTTTGTCAACTGGAAAACAGGCGATCCCTCTGGTGAACGGAATGATTGT
*Msr1*	NM_001113326.1	GATTTCGTCAGTCCAGGAACATGGGAATTCACTGGATGCAATCTCCAAGTCCTTGCAGAGTCTGAATATGACACTGCTTGATGTTCAACTCCATACAGAA
*Myc*	NM_010849.4	CCCTCAACGTGAACTTCACCAACAGGAACTATGACCTCGACTACGACTCCGTACAGCCCTATTTCATCTGCGACGAGGAAGAGAATTTCTATCACCAGCA
*Ncf1*	NM_001286037.1	ACCATCCGCAACGCACAGAGCATCCACCAGCGTTCTCGGAAGCGCCTTAGCCAGGACACCTATCGCCGCAACAGCGTCCGATTCCTGCAGCAGCGCAGAC
*Nfkbia*	NM_010907.1	GTCAGAATTCACAGAGGATGAGCTGCCCTATGATGACTGTGTGTTTGGAGGCCAGCGTCTGACATTATAAGTGGAAAGTGGCAAAAAAGAATGTGGACTT
*Nos2*	NM_010927.3	CCCCCCTCCTCCACCCTACCAAGTAGTATTGTACTATTGTGGACTACTAAATCTCTCTCCTCTCCTCCCTCCCCTCTCTCCCTTTCCTCCCTTCTTCTCC
*Nox1*	NM_172203.1	CTCCAAACATGACAGTGATGTATGCAGCATTTACCAGTATTGCTGGCCTTACTGGAGTGATTGCCACTGTAGCTTTGGTTCTCATGGTAACGTCAGCTAT
*Nox4*	NM_015760.4	TCCCAGAAAGCTTCTCTTCACAACCATTCCTGGTCTGACGGGTGTCTGCATGGTGGTGGTATTGTTCCTCATGGTTACAGCTTCTACCTACGCAATAAGA
*Nr3c1*	NM_008173.3	ACCAGGATTCAGAAACTTACACCTGGATGACCAAATGACCCTTCTACAGTACTCATGGATGTTTCTCATGGCATTTGCCCTGGGTTGGAGATCATACAGA
*P2rx7*	NM_001038887.1	CTGGAGGAACTGGAAGTTAACCGTTCCTGCTGAGAAATCGGTGTGTTTCCTTTGGCTGCTCCTAGGTGAGGGTTTGCTGTGGTCTAGCCTGGGAAGTAGG
*P2ry12*	NM_027571.3	GATCACCCAGGTTCTCTTCCCATTGCTGTACACCGTCCTGTTCTTTGCTGGGCTCATCACGAACAGCTTGGCAATGAGGATTTTCTTTCAGATCCGCAGT
*P2ry2*	NM_008773.3	TAGCCATTTTGTGGCTTACAGCTCCGTCATGCTGGGTCTGCTTTTTGCTGTGCCCTTTTCCGTAATCCTGGTCTGTTACGTGCTTATGGCCAGGCGGCTG
*Pparg*	NM_011146.1	ACCAAGTGACTCTGCTCAAGTATGGTGTCCATGAGATCATCTACACGATGCTGGCCTCCCTGATGAATAAAGATGGAGTCCTCATCTCAGAGGGCCAAGG
*Ptgs2*	NM_011198.3	CCATCAGTTTTTCAAGACAGATCATAAGCGAGGACCTGGGTTCACCCGAGGACTGGGCCATGGAGTGGACTTAAATCACATTTATGGTGAAACTCTGGAC
*Ptk2b*	NM_001162365.1	CTTCCGCCGCTTCACAACCGCCAGTGATGTCTGGATGTTTGCTGTATGCATGTGGGAGATCCTCAGCTTTGGGAAGCAGCCTTTCTTCTGGCTCGAAAAT
*Ptpn6*	NM_013545.2	GACCGAGGCCCAGTACAAGTTTATTTACGTGGCCATTGCCCAGTTCATCGAAACGACCAAGAAGAAACTGGAGATCATACAATCCCAGAAGGGCCAGGAG
*Retnla*	NM_020509.3	GAATACTGATGAGACCATAGAGATTATCGTGGAGAATAAGGTCAAGGAACTTCTTGCCAATCCAGCTAACTATCCCTCCACTGTAACGAAGACTCTCTCT
*Socs1*	NM_009896.2	CAGCTTGTGTCTGGGGCCAGGACCTGAATTCCACTCCTACCTCTCCATGTTTACATATTCCCAGTATCTTTGCACAAACCAGGGGTCGGGGAGGGTCTCT
*Socs3*	NM_007707.2	CCGCGACAGCTCGGACCAGCGCCACTTCTTCACGTTGAGCGTCAAGACCCAGTCGGGGACCAAGAACCTACGCATCCAGTGTGAGGGGGGCAGCTTTTCG
*Tgfb1*	NM_011577.1	GGAGTTGTACGGCAGTGGCTGAACCAAGGAGACGGAATACAGGGCTTTCGATTCAGCGCTCACTGCTCTTGTGACAGCAAAGATAACAAACTCCACGTGG
*Tgfbr1*	NM_009370.2	TCAGAAGTAGTGGCCAGCTGTGTCTCTAGTAGGACAGTAAAGGCATGAAGCTCAGCCTGTAATCCTGCTACTACAGTAGTACTCCAGAAGTGCCTTGAGG
*Tgfbr2*	NM_009371.2	TGTGCAAGTTTTGCGATGTGAGACTGTCCACTTGCGACAACCAGAAGTCCTGCATGAGCAACTGCAGCATCACGGCCATCTGTGAGAAGCCGCATGAAGT
*Tlr2*	NM_011905.2	GCAGGCGGTCACTGGCAGGAGATGTGTCCGCAATCATAGTTTCTGATGGTGAAGGTTGGACGGCAGTCTCTGCGACCTAGAAGTGGAAAAGATGTCGTTC
*Tlr4*	NM_021297.2	AACGGCAACTTGGACCTGAGGAGAACAAAACTCTGGGGCCTAAACCCAGTCTGTTTGCAATTAATAAATGCTACAGCTCACCTGGGGCTCTGCTATGGAC
*Tnf*	NM_013693.1	TTCCTGAGTTCTGCAAAGGGAGAGTGGTCAGGTTGCCTCTGTCTCAGAATGAGGCTGGATAAGATCTCAGGCCTTCCTACCTTCAGACCTTTCCAGACTC
*Tnfrsf1a*	NM_011609.2	CTCCTTGCCAAGCTGACAAGGACACGGTGTGTGGCTGTAAGGAGAACCAGTTCCAACGCTACCTGAGTGAGACACACTTCCAGTGCGTGGACTGCAGCCC
*Tnfrsf1b*	NM_011610.3	GTGTGTGTCCATGTTTGCATGTATGTGTGTGCCAGTGTGTGGAGGCCAGAGGTTGGCTTTGGGTGTGTTTGATCACTCTCAGTTACTGAGGCAGGGCTCT
*Trem2*	NM_031254.2	GGGCGCCTACCCTAGTCCTGACTGTTGCTCAATCCAGGAGCACAGTTCCTGTGGGCTGAGCCTGACTGGCTTGGTCATCTCTTTTCTGCACTTCAAGGGA
*Tspo*	NM_009775.4	GACACTGGCTCCCATCTGGGGCACACTGTATTCAGCCATGGGGTATGGCTCCTACATAGTCTGGAAAGAGCTGGGAGGTTTCACAGAGGACGCTATGGTT

We analyzed over 50 genes in rat and mouse microglia under different activation states. To assess microglial activation, markers, pro- and anti-inflammatory mediators, receptors and signaling molecules were analyzed. We also assessed several immunomodulators, nicotinamide adenine dinucleotide phosphate-oxidase (NOX) enzymes, purinergic and phagocytic receptors, and potassium (K^+^) channels that play roles in microglial functions. For inter-species comparisons, we converted relative mRNA counts to fold changes relative to unstimulated (control) levels and then compared fold changes in response to cytokine stimulation.

### Western blot analysis

#### General methods

Microglia were seeded on 25-mm coverslips in 35-mm cultures dishes. Rat pups yielded much higher microglial numbers; i.e., three rat pups from a single litter provided enough to seed at 1–3 × 10^6^ cells, and for all four treatments (CTL, I + T, IL-4, IL-10). For mouse, we had to combine microglia from 5 to 6 entire litters in order to seed at ~5 × 10^5^ cells, which was the minimum needed for a single Western blot, and for only two treatments; i.e., CTL and I + T or CTL and IL-4. Because of this limitation on mouse microglial numbers, and the minimal effects of IL-10 on rat microglia, we did not treat mouse cells with IL-10. In addition, the number of individual replicates was smaller for mouse (*n* = 3–7) than rat (*n* = 14–22).

After stimulating for 24 h, microglial cells were briefly washed with PBS. The cells were lysed for 30 min in ice-cold RIPA buffer with a protease inhibitor cocktail designed for use with mammalian cell and tissue extracts (Sigma-Aldrich, Oakville, ON, Canada), and then spun down to pellet insoluble material. The total protein concentration in the lysates was determined using the Pierce™ BCA protein assay (ThermoFisher Scientific, Mississauga, ON, Canada). Lysates were stored at −80 °C until used. Before SDS-PAGE, proteins were denatured (100 °C for 5 min in a dry-bath incubator) in NuPage LDS sample buffer (Thermofisher) with 5% 2-β-mercaptoethanol. Samples were loaded on 8 or 12% acrylamide gels at 10 μg protein/lane and electrophoresed for 1.5–2 h at a constant voltage of 80 mV while the samples ran through stacking gel, and 120 mV through the resolving gel. After transferring proteins to a PVDF membrane at 100 mA for 1.5 h, membranes were blocked in 5% non-fat dry milk in Tris-Tween buffered saline (TTBS) for 2–3 h. Membranes were incubated on an orbital shaker overnight at 4 °C in primary antibodies diluted in TTBS containing 1% bovine serum albumin (BSA). The antibodies and concentrations were mouse anti-glyceraldehyde-3-phosphate dehydrogenase (GAPDH; 1:10,000), rabbit anti-α-tubulin (1:5000), mouse anti-inducible nitric oxide synthase (iNOS/NOS2, 1:250), rabbit anti-protein tyrosine kinase 2 beta (PYK2, 1:500), rabbit anti-arginase1 (ARG1, 1:2000), rabbit anti-ionized calcium-binding adapter molecule 1 (Iba1, 1:500), rabbit anti-cyclooxygenase 2 (COX-2, 1:1000), or rabbit anti-mannose receptor (MRC1/CD206, 1:2000). Primary antibodies were from Abcam (Cambridge, MA), except for anti-GAPDH (EMD Millipore, Etobicoke, ON, Canada) and anti-Iba1 (Wako Chemicals, Richmond, VA). The next day, membranes were washed (3 × 10 min) in 1% BSA-TTBS and incubated in horseradish peroxidase-conjugated goat anti-rabbit or mouse IgG antibodies (1:3000 in 1% BSA-TTBS; Cedarlane, Burlington, ON, Canada) for 1 h at room temperature. After repeated washing (6 × 3 min), protein bands were visualized using Amersham ECL Start Western Blotting Detection Reagents (GE Healthcare Life Sciences, Mississauga, ON, Canada), using the ChemiDoc™ XRS System (Bio-Rad, Mississauga, ON, Canada).

#### Protein normalization

The housekeeping proteins, α-tubulin or GAPDH, are often used to normalize Western blots. However, we found that GAPDH protein increased after I + T treatment, especially in rat, and that α-tubulin protein was much higher in rat microglia than mouse, and it increased with IL-4 treatment (not shown). Therefore, we used the more recently recommended approach of total protein normalization using Coomassie staining of immunoblots [46]. After immunodetection, membranes were stained with 0.1% Coomassie Brilliant Blue G (Sigma-Aldrich) for 1 min, de-stained for 10 min in acetic acid/methanol/water (1:5:4), air-dried, and imaged using the ChemiDoc™ XRS System. Densitometry analysis was conducted using Image Lab ver.5.2.1(Bio-Rad). Band density is defined as the volume of the user-delineated lane and band of interest and the chemiluminescent signal detected by the ChemiDoc System. If the protein of interest was lower than visually apparent (e.g., control levels of iNOS), a band of the same size and location was measured by the software. After subtracting background, intensities of protein bands of interest were normalized to the total Coomassie blue staining intensity of a given lane. In a pilot study on rat microglia, the coefficient of variability across all treatments was 36% (±15% SD) for GAPDH and only 18% (±7% SD) for Coomassie blue staining. The normalized intensity of a protein of interest was then expressed as fold change relative to unstimulated (CTL) cells. Many samples were run in duplicate or triplicate on different gels, which allowed average fold changes for the single biological replicate to be used in the statistical analysis.

### Nitric oxide production

Microglia were seeded at 8 × 10^4^ per glass coverslip and were either left unstimulated or treated with I + T, IL-4 or IL-10 for 24 h. The colorimetric Griess assay (Invitrogen) was used to measure nitrite levels as an indirect measure of nitric oxide production. For the Griess reaction, 200 μl of conditioned medium from microglia samples was added to wells of a 96-well plate containing 25 μl sulfanilic acid. Then, 25 μl 0.1% N-(1-naphthyl) ethylenediamine was added, and the medium was kept in the dark at room temperature for 30 min to allow the reaction to occur. The color change in the samples was quantified using a multi-label plate reader (Victor^3^ 1420, Perkin Elmer, Woodbridge, ON, Canada) set at an absorbance wavelength of 570 nm. Nitrite concentrations in the samples were determined by interpolation on a standard curve generated from a series of NaNO_2_ samples of known concentration. Results are expressed as fold change relative to untreated (CTL) samples.

### Expression of K^+^ channels and currents

#### Quantitative real-time reverse-transcriptase polymerase chain reaction (qRT-PCR)

Expression of Kir2 subfamily members was assessed in unstimulated and stimulated rat and mouse microglia. qRT-PCR primers were designed using “Primer3web” (http://bioinfo.ut.ee/primer3/) to detect the genes encoding *Kcnj2* (Kir2.1): forward (5′-ACCGCTACAGCATCGTCTCT-3′) and reverse (5′-CTGCACTGTTGTCGGGTATG-3′); *Kcnj12* (Kir2.2): forward (5′- AACCCCTACAGCATCGTATC-3′) and reverse (5′- GCACCTTGCCATTGCCAAA-3′); *Kcnj4* (Kir2.3): forward (5′-AACAAGTCCCAGCGCTACATG-3′) and reverse (5′-AGGAAGGCCGCGGAGAAG-3′); and *Kcnj14* (Kir2.4): forward (5′-AGTGCATCGCAGGCT GTGTG-3′) and reverse (5′-CACTGCGTTCTCACTGAAGAC-3′). Primers for the housekeeping gene, *Hprt1*, were: forward (5′-CAGTACAGCCCCAAAATGGT-3′) and reverse (5′- CAAGGGCATATCCAACAACA-3′). Extracted RNA (0.25 μg) was reverse transcribed using SuperScriptII RNase reverse transcriptase, with dNTPs, oligo dT, and DTT (according to instructions from Invitrogen). cDNA was then amplified using an ABI PRISM 7700 Sequence Detection System (PEBiosystems, Foster City, CA, USA), with the following protocol: 50 °C for 2 min, 95 °C for 10 min, 40 cycles at 95 °C for 15 s, 60 °C for 60 s, and three dissociation steps (95 °C for 15 s, 60 °C for 15 s, 95 °C for 15 s). The threshold cycle (CT) for each member of the Kir2 family was normalized to *Hprt1* (∆CT) and converted to 2^∆CT^.

#### Whole-cell patch-clamp recordings

For each assay, a coverslip bearing unstimulated or stimulated rodent microglia (7–9 × 10^4^ cells/coverslip) was mounted in a 300-μL volume perfusion chamber (Model RC-25, Warner Instruments, Hamden, CT). The standard bath solution consisted of (in mM) 125 NaCl, 5 KCl, 1 CaCl_2_, 1 MgCl_2_, 10 HEPES, 5 D-glucose, adjusted to pH 7.4, and 290–300 mOsm/kg H_2_O. Bath solution, with or without a channel blocker, was perfused into the chamber using a gravity-driven perfusion system flowing at ~1 mL/min. Recording pipettes (5–8 MΩ resistance) were pulled from thin wall borosilicate glass (WPI, Sarasota, FL) on a Narishige puller (Narishige Scientific, Setagaya-Ku, Tokyo), and fire polished with a microforge (MF 900; Narishige). Pipettes were filled with an intracellular solution containing (in mM) 40 KCl, 100 KAsp, 1 MgCl_2_, 10 HEPES, and 2 MgATP (pH 7.2; 290–300 mOsm/kg H_2_O) and with 0.5 CaCl_2_ and 1 EGTA to buffer internal free Ca^2+^ to ~120 nM. Data were acquired using an Axopatch 200A amplifier, filtered at 5 Hz with a DigiDATA 1322A board, and analyzed with pCLAMP 10 software (all from Molecular Devices, Sunnyvale, CA). The ground electrode was inserted into an agar bridge made with bath solution in order to reduce junction potentials, which were then calculated with the pCLAMP utility. All nominal voltages were shifted by **−**15 mV to account for the junction potential (**−**12.6 mV) and headstage leak as indicated in the voltage protocols, figure legends, and “Results” text.

Kv1-family members are activated by depolarization, but because they also undergo inactivation during sustained or repetitive depolarizing pulses, the current amplitude depends on the holding potential, test potential, and frequency of depolarization. The voltage dependence of activation and steady-state inactivation can also be modulated (e.g., by phosphorylation, as shown for Kv1.3 [47–49]), so it is crucial to quantify the current over a range of potentials. In addition, a hallmark of Kv1.3 is cumulative inactivation, which is seen as a use-dependent decrease in current if successive depolarizing pulses are delivered too soon [28, 50–52]. For rat microglia, substantial cumulative inactivation is evoked by repetitive pulses every 1 s [52] or 5 s [28, 50], while an interpulse interval of 60 s ensures complete recovery from inactivation [28]. Therefore, to quantify Kv currents in rat microglia, we used a holding potential of −105 mV to relieve channel inactivation, and used 60-s intervals between successive depolarizing steps. The entire rat protocol required ~20 min per recording. Because recordings from mouse microglia did not usually last as long, it was necessary to modify the protocol. A voltage ramp from −75 to +45 mV was applied from the −105-mV holding potential, after the protocol was validated by ensuring that the amplitude at +45 mV was the same as for a single voltage step.

Agitoxin-2 (AgTx-2) is an extremely potent Kv1.3 blocker [53], with a *K*_*d*_ of 177 pM in activated T lymphocytes [28]. To quantify the Kv1.3 component, 5 nM AgTx-2 (Sigma-Aldrich) was perfused into the bath, and the remaining unblocked current was subtracted. For both patch-clamp recordings and functional assays (transmigration, proliferation), AgTx-2 was used to block Kv1.3 and ML133 (Sigma-Aldrich) was used to block Kir2.1 channels. Stock solutions were prepared in dimethyl sulfoxide (Tocris Bioscience, MO) for ML133, and in double-distilled water with 0.02% BSA for AgTx-2, and then aliquoted and stored at −20 °C until used. Inhibitor solutions were diluted to working concentrations of 20 μM ML133 and 5 nM AgTx-2.

### Microglia staining and transmigration assay

Microglia were seeded at 7–9 × 10^4^ cells/coverslip, and stimulated with cytokines for 24 h. They were briefly washed in phosphate-buffered saline (PBS), and fixed in 4% paraformaldehyde (Electron Microscopy Sciences, Hatfield, PA) at room temperature for 15 min. After permeabilizing the cells with 0.2% Triton X-100 for 5 min, they were washed in PBS (3×, 5 min), and labeled with Alexa Fluor 488-conjugated phalloidin (1:50 in PBS for 1 h; Invitrogen) to visualize filamentous (F-) actin, and with 4′,6-diamidino-2-phenylindole (DAPI; 1:3000 in PBS for 10 min; Invitrogen) to label nuclei. After washing (3×, 5 min), coverslips were mounted on glass slides using Dako mounting medium (Dako, Glostrup, Denmark) and stored in the dark at 4 °C. Images were acquired using an Axioplan 2 wide-field epifluorescence microscope equipped with an Axiocam HR digital camera (both from Zeiss, Toronto, ON, Canada).

To quantify migration, microglia were seeded at 3 × 10^4^ cells per insert filter (which bore 8-μm-diameter holes), and placed in the upper well of a Transwell migration chamber (VWR, Mississauga, ON, Canada) containing 500 μL MEM with 2% FBS, as recently described [18, 20, 30]. After 30 min, 500 μL of MEM with 2% FBS was added to the lower well, and microglia were left unstimulated or stimulated for 24 h (37 °C, 5% CO_2_) with I + T, IL-4, or IL-10, as above. When used, a channel blocker (ML133 or AgTx-2) was added at the time of cytokine addition. Transwell inserts were then briefly washed with PBS, fixed for 10 min in 4% paraformaldehyde, and washed again in PBS (3×, 5 min). A Q-tip was used to remove microglia from the top of the Transwell inserts. Cells that had migrated to the underside of the membrane were stained with 0.3% crystal violet in methanol (~1 min) and washed with PBS. Cells from five random fields at 40× magnification were counted using an Olympus CK2 inverted microscope (Olympus, Tokyo), summed and normalized to the unstimulated (CTL) group.

### Proliferation

We used the CyQuant NF assay (Invitrogen) to measure cell proliferation, as previously described [18, 30]. Microglia were seeded at 2–3 × 10^4^ cells per well of a 96-well flat-bottom plate and cultured in MEM with 2% FBS for 1–2 days. Then, cells were unstimulated or stimulated with I + T, IL-4, or IL-10 in the presence or absence of a channel blocker (ML133 or AgTx-2). After 24 h, the CyQuant dye solution was added to each well and incubated for 30 min (37 °C, 5% CO_2_). The fluorescence intensity was measured using a multi-label plate reader (Victor^3^ 1420, Perkin Elmer, Woodbridge, ON, Canada), with excitation at 485 nm and emission at 535 nm. Readings were taken for 0.1 s at 3 mm from the bottom of the plate in duplicate and were averaged, and background was subtracted before normalizing to the unstimulated (CTL) group.

### Statistics

All graphical data are presented as mean ± SEM for the number of replicates indicated. The statistical significance was analyzed in GraphPad ver 6.01 (GraphPad Software, San Diego, CA) using either a one-way analysis of variance (ANOVA) with Dunnett’s post hoc analysis or two-way ANOVA with Bonferroni post hoc analysis (electrophysiology, Western blotting, Griess and migration assays). For NanoString analysis, the mRNA counts acquired after normalization were expressed as fold changes relative to control cells to compare the effects of activation responses between rat and mouse microglia. A two-way ANOVA with Fisher’s LSD test was then conducted, and the *p* values for differences in activation state or species were adjusted using a 5% false discovery rate correction for multiple comparisons [54] in R (version 3.3.1).

## Results

### Inflammatory profiling of rat and mouse microglia

The terminology for microglial activation is evolving; thus, for clarity, activation states are denoted by the stimulus used, as follows. *M*(*I + T*)*.* Microglia were treated with a combination of IFN-γ and TNF-α to evoke a pro-inflammatory state (also called classical or M1 activation), as before [17]. *M*(*IL-4*)*.* IL-4 was applied to skew them toward an anti-inflammatory state (also called alternative or M2a activation). *M*(*IL-10*)*.* IL-10 was used to skew them toward an acquired deactivation state (sometimes called M2c).

Those results showed increases in pro-inflammatory markers after LPS treatment (e.g., NOS2, TNFα, IL-1β) versus increases in anti-inflammatory markers after IL-4; e.g., arginase 1 (ARG1), CD163, mannose receptor (MRC1/CD206), IL-4 receptor α (IL-4RA), IL-10, and TGF-β1 [19, 30, 35]. In addition, our previous NanoString analysis showed that hallmark M1 and M2a responses could be detected in vitro and in vivo [17, 55]. Here, to create a comparison profile of responses of rat and mouse microglia, transcript expression was quantified by NanoString for 58 genes encompassing pro- and anti-inflammatory mediators and their receptors, other immunomodulators (nicotinamide adenine dinucleotide phosphate-oxidase (NOX) enzymes), and purinergic and phagocytic receptors. The gene nomenclature is indicated for rat (Table 1) and mouse (Table 2); however, for simplicity, the rat names will be used.

To illustrate differences in basal transcript abundance, the left-hand columns of Tables 3, 4, 5, and 6 show results for each gene, stated as the number of mRNA counts per 200 ng sample under control (unstimulated) conditions. Then, for M(I + T), M(IL-4), and M(IL-10) stimulation paradigms, results are shown as fold changes with respect to the control values. Significant differences within a species are indicated by up arrows for increases and down arrows for decreases. In addition, these tables indicate species differences within an activation paradigm (bold numbers and asterisks). [Additional files 1, 2, 3, 4, and 5: Fig. S1–S5, graphically show the complete mRNA data for both species.]

#### Pro-inflammatory genes and receptors. Unstimulated

As previously shown for rat microglia [17, 19, 56], unstimulated (control; CTL) microglia from both rodent species were in a relatively resting state, exemplified here by very low transcript levels (<100 mRNA counts/200 ng sample) of several pro-inflammatory mediators (*Nos2*, *Il6*, *Ptgs2* (COX-2), *Ifng* (IFN-γ), and the IL-1β receptor (*IL1r1*) (Table 3; Additional file 1: Fig. S1). Both species expressed similar, moderate levels (>200 counts) of *Casp1* (caspase-1, ICE), the protein kinase *Ptk2b* (proline-rich tyrosine kinase 2, PYK2), *Tnf*, and the TNF-α receptor, *Tnfrsf1a* (TNFR1). The main species differences in control cells were very low *Ifngr2* expression in rat but a moderate level in mouse, and very low IL1β in mouse versus a moderate level in rat. Although the TNF-α receptor, *Tnfrsf1b* (TNFR2), and the IFN-γ receptor, *Ifngr1*, were moderately expressed in both species, they were 3.1-fold and 3.4-fold higher in control rat microglia, respectively. *M*(*I + T*)*.* We confirmed that I + T stimulation skews rat microglia toward a pro-inflammatory state [17], with elevated *Nos2*, *Tnf*, and *Il6* expression. In rat cells, *Ptk2b*, *Tnfrsf1a*, and *Tnfrsf1b* were also elevated, while transcript expression was unchanged for *Ifng* and its receptors (*Ifngr1*, *Ifngr2*) and for *Il1b*, its receptor (*Il1r1*), *Casp1*, and *Ptgs2*. [A pilot NanoString analysis of rat microglia at 6 h after I + T treatment showed significant increases in *Nos2*, *Tnf*, and *IL1b* (data not shown).] In mouse microglia, I + T significantly increased many of the same pro-inflammatory genes, but differences (bold numbers) were the increases in *Ifng*, *Ifngr2*, *Il1r1*, and *Casp1*, and lack of change in *Tnfrsf1b*. Other species differences were the greater induction of *Nos2* (4.7-fold higher) and *Ptk2b* (5.1-fold higher) in rat, while mouse showed a greater induction of *Tnf* (3.2-fold) and *Tnfrsf1a* (1.5-fold). Induction of *Ptgs2* by I + T was also greater in mouse cells (8.4-fold), and although it did not reach statistical significance, rat cells showed a nearly 26-fold increase (*p* = 0.07). *M*(*IL-4*)*.* IL-4 did not induce expression of pro-inflammatory genes in either species. Instead, *Ifngr1* decreased in mouse only and *Tnfrsf1b* showed opposite changes in expression in the two species (down in rat, up in mouse). *M*(*IL-10*)*.* There were no changes in pro-inflammatory transcript levels, except for an increase in *Il1b* (mouse only).

**Table 3:** Transcript expression of pro-inflammatory genes and receptors

	Control		I + T		IL-4		IL-10	
	Relative RNA counts ± SD		Fold change with respect to Control					
	Rat	Mouse	Rat	Mouse	Rat	Mouse	Rat	Mouse
*Casp1* (ICE)	797 ± 180	849 ± 114	1.37	**6.88** ^↑↑↑ ***^	0.87	1.14	1.14	1.06
*Ifng*	6 ± 4	2 ± 2	1.19	**3.71** ^↑*^	0.64	1.28	0.69	1.61
*Ifngr1*	8056 ± 1346	2405 ± 235	1.18	0.91	0.74	0.61 ^↓↓^	1.11	1.07
*Ifngr2*	30 ± 11	1646 ± 174	1.36	**1.75** ^↑↑↑ *^	1.23	1.18	1.11	1.11
*Il1b*	1524 ± 1011	47 ± 27	1.21	2.36	0.23	0.18	1.31	**4.86** ^↑↑↑ ***^
*Il1r1*	12 ± 5	11 ± 5	1.57	2.15 ^↑^	1.55	1.67	0.88	1.14
*Il6*	10 ± 6	9 ± 2	3.33 ^↑↑↑^	3.24 ^↑↑^	0.98	0.88	0.79	0.55
*Nos2* (iNOS)	44 ± 33	24 ± 19	**1432.35** ^↑↑↑ ***^	306.45 ^↑↑↑^	0.37	2.48	1.70	1.25
*Ptgs2* (COX-2)	22 ± 17	54 ± 27	25.88	**217.33** ^↑↑↑ ***^	1.76	3.55	1.34	1.08
*Ptk2b* (PYK2)	1386 ± 299	931 ± 130	**10.97** ^↑↑↑ ***^	2.14 ^↑↑↑^	0.70	0.89	1.48	1.05
*Tnf* (TNF-α)	511 ± 214	220 ± 129	3.91 ^↑↑↑^	**12.42** ^↑↑↑ ***^	0.38	0.62	1.08	0.50
*Tnfrsf1a* (TNFR1)	1107 ± 46	836 ± 40	3.05 ^↑↑↑^	**4.60** ^↑↑↑ **^	1.00	1.06	1.44	1.59
*Tnfrsf1b* (TNFR2)	1915 ± 109	612 ± 83	**2.37** ^↑↑↑ ***^	1.35	0.60	1.49 ^**^	1.28	0.98

Rat and mouse microglia were unstimulated (CTL) or stimulated with IFN-γ plus TNF-α (I + T), IL-4 or IL-10 for 24 h. mRNA counts for each gene were normalized to two housekeeping genes (see Methods). For clarity, protein names are included for some genes. To show differences in basal mRNA levels, unstimulated (control) counts are expressed as mean ± SD (*n* = 4–6 individual cultures). Effects of activation state on a given gene are expressed as fold changes relative to species-matched control levels. Arrows indicate statistically significant increases (↑) or decreases (↓) in expression. Species differences within an activation paradigm are indicated by bold numbers and asterisks. One symbol (arrow or asterisk) indicates *p* < 0.05; two, *p* < 0.01; three, *p* < 0.001; four, *p* < 0.0001

**Fig. 1:**
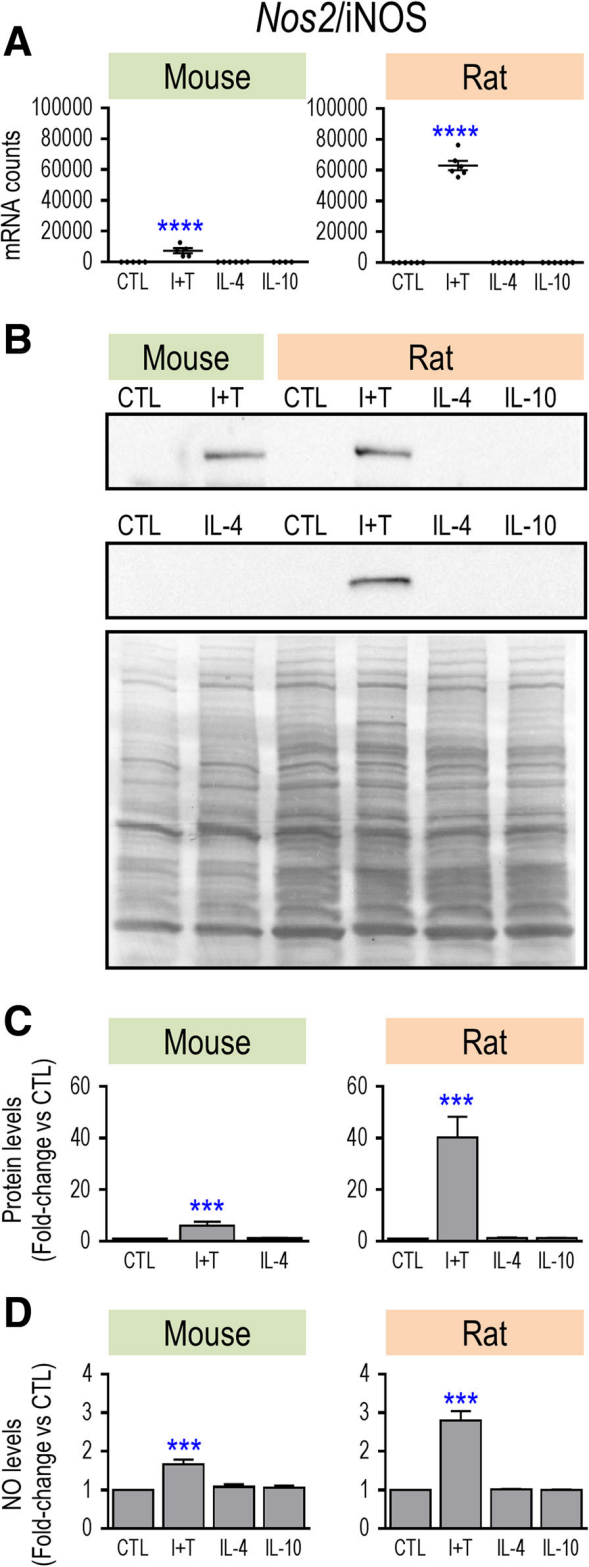
Species comparison of *NOS2* mRNA, iNOS protein, and nitric oxide production. Microglia were unstimulated (CTL) or stimulated with IFN-γ plus TNF-α (I + T), IL-4, or IL-10 for 24 h. **a** *NOS2* mRNA expression (mRNA counts/200 ng total RNA) was determined by NanoString. mRNA counts for each gene were normalized to two housekeeping genes (described in Methods) and are shown as mean ± SEM (*n* = 4–6 individual cultures), plotted on the same *Y*-axis scale. **b** Two representative Western blots of iNOS protein, with both species on the same gel. For each example, the full membrane was stained with Coomassie blue (lower panel), which was used to normalize iNOS protein levels. **c** Summary of fold changes in iNOS protein (mean ± SEM; *n* = 4–6 individual cultures for mouse and 22 for rat). For each Western blot, each iNOS band was normalized to total protein in that lane and then, iNOS levels for each treatment were normalized to unstimulated (control) microglia. **d** Nitric oxide production was monitored using the Griess assay (mean ± SEM; *n* = 6–11 individual cultures). Significant differences from unstimulated (control) cells are indicated: ***p < 0.001; ****p < 0.0001

**Fig. 2:**
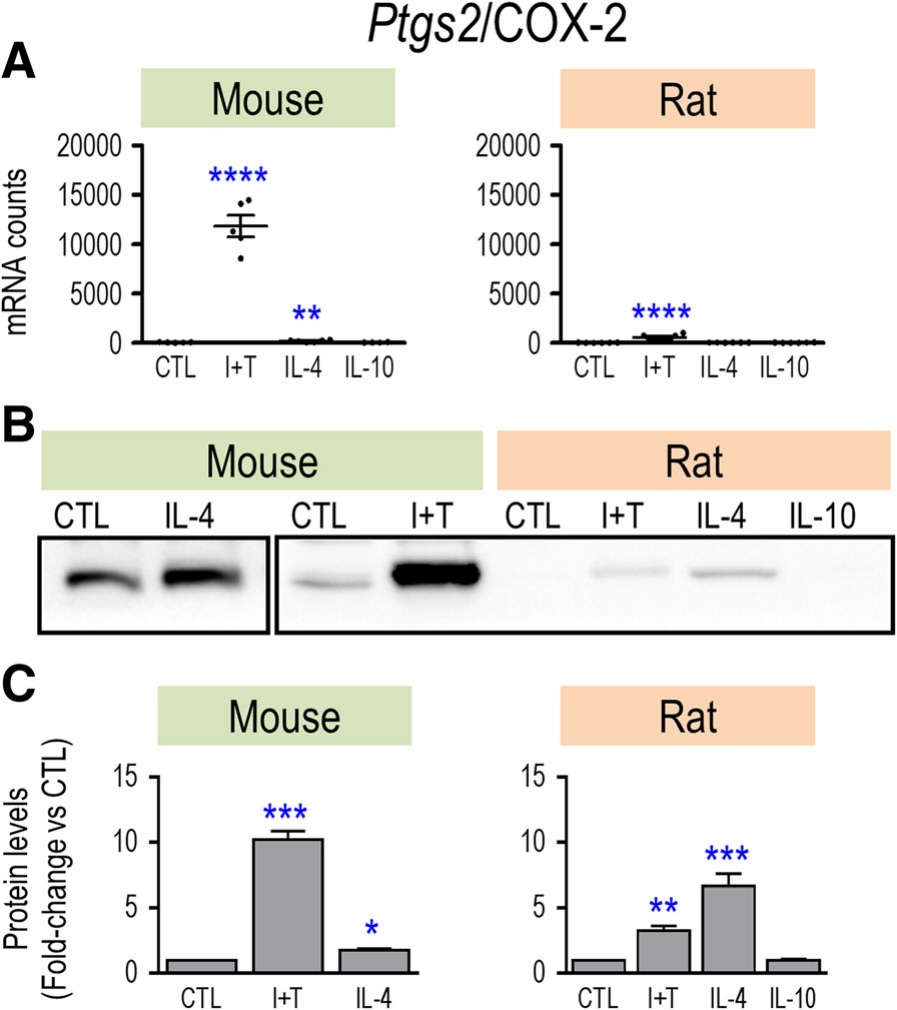
Species comparison of *Ptgs2* mRNA and COX-2 protein. Treatments and data presentation are as in Fig. 1. **a** *Ptgs2* mRNA expression (mRNA counts/200 ng total RNA) was determined by NanoString analysis (mean ± SEM; *n* = 4–6 individual cultures), and plotted on the same *Y*-axis scale. **b** Representative Western blots of COX-2 protein. **c** Summary of fold changes in COX-2 protein expression (mean ± SEM; *n* = 4–6 individual cultures for mouse and 16 for rat), normalized to the total protein in each lane, and then to control microglia as in Fig. 1. Significant differences from unstimulated (control) cells are indicated: *p < 0.5; **p < 0.01; ***p < 0.001; ****p < 0.0001

**Fig. 3:**
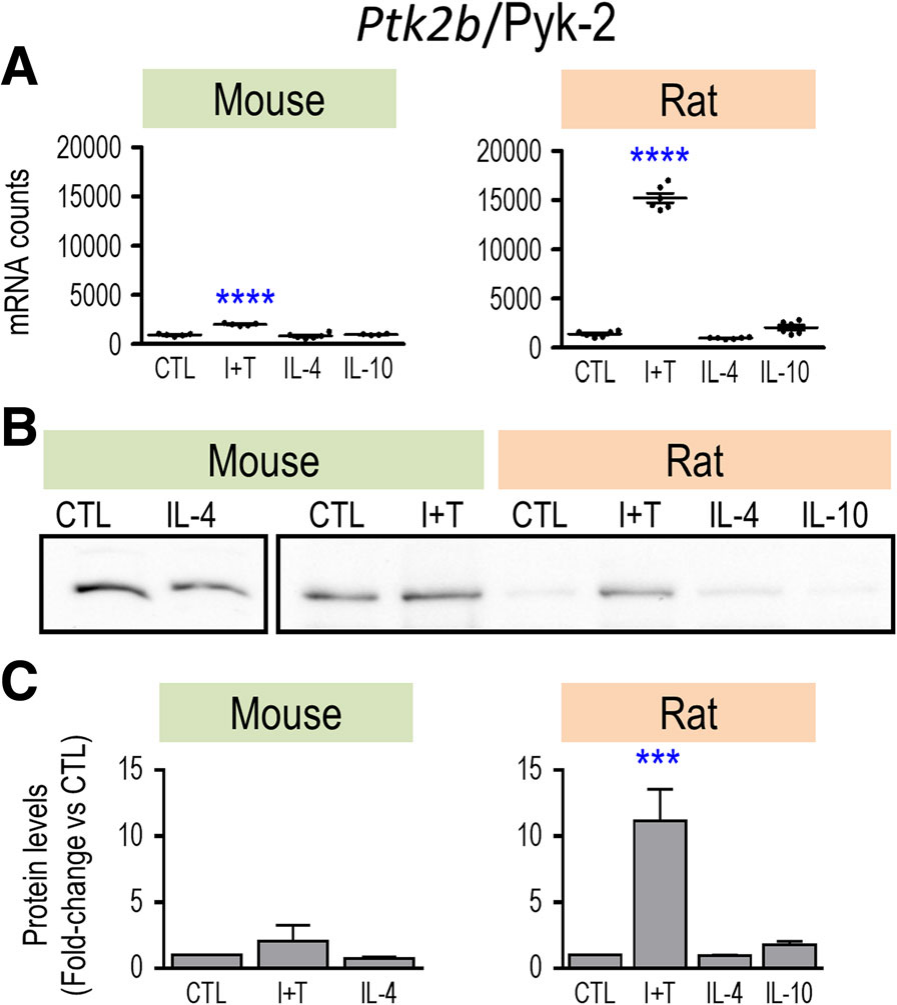
Species comparison of *Ptk2b* mRNA and Pyk2 protein. Treatments and data presentation are as in Fig. 1. **a** *Ptk2b* mRNA expression (mRNA counts/200 ng total RNA) was determined by NanoString analysis (mean ± SEM; *n* = 4–6 individual cultures), and plotted on the same *Y*-axis scale. **b** Representative Western blots of Ptk2 protein. **c** Summary of fold changes in Pyk2 protein expression (mean ± SEM; *n* = 4–6 individual cultures for mouse and 22 for rat), normalized to the total protein in each lane, and then to control microglia as in Fig. 1. Significant differences from unstimulated (control) cells are indicated: ***p < 0.001; ****p < 0.0001

Overall, only M(I + T) cells had increased mRNA expression of common pro-inflammatory genes. However, because quantitative differences were seen between rat and mouse, we next used Western analysis to examine protein changes for some key molecules. For iNOS, both mRNA and protein were induced, and only by I + T in both species (Fig. 1). Importantly, species differences seen in the magnitude of Nos2 mRNA counts after I + T treatment (Fig. 1a) were reflected by differences in iNOS protein upregulation; i.e., 40-fold increase in rat versus 5.4-fold in mouse (Fig. 1b, c). Consistent with these changes, the I + T-induced increase in nitric oxide production was 2.8-fold in rat cells versus 1.7-fold in mouse cells (Fig. 1d). Thus, a species difference in the magnitude of response was seen at every level: mRNA, protein, and functional outcome. A different pattern was seen for Ptgs2/COX-2. I + T treatment increased both Ptgs2 mRNA (Fig. 2a) and COX-2 protein (Fig. 2b, c) in both species but increases in both mRNA and protein were much higher in mouse. IL-4 treatment also increased COX-2 protein in both species. Ptk2b/PYK2 showed interesting species similarities and differences. Based on mRNA counts, Ptk2b appeared to be a good M1 marker, as it was induced only by I + T, and in both species (Fig. 3a), although the level was much higher in rat (~15,000 vs ~2000 counts/200 ng sample). The resting level of PYK2 protein appeared to be lower in rat (Fig. 3b), and I + T significantly increased it in rat cells only (Fig. 3b, c).

#### Anti-inflammatory and “alternative” activation genes and receptors

We examined several genes known to be upregulated by IL-4 in mouse microglia: ARG1, “found in inflammatory zone” 1 (FIZZ1), MRC1/CD206, CCL22, CD163, and peroxisome proliferator-activated receptor gamma (PPAR-γ) [57]*.* To further investigate anti-inflammatory responses, we also examined IL-4, IL-10, TGF-β1, and their receptors*. Unstimulated.* Both species had low transcript levels (<100 counts/200 ng sample) of the alternative-activation markers, *Arg1*, *Ccl22*, *Retnla* (FIZZ1), and *Cd163*, as well as *Il4* and *Il10* (Table 4; Additional file 2: Fig. S2 and Additional file 3: Fig. S3). Both species expressed similar, moderate to high levels of *Mrc1*, *Myc*, and the receptors, *Tgfbr2*, *Il10ra*, *Il10rb*, and *Il13ra1*. Rat cells had higher transcript levels of *Tgfb1* (6.3-fold), *Tgfbr1* (3.8-fold), *Il1rn* (8.4-fold), *Il4r* (4.2-fold), and *Pparg* (8.6-fold). *M*(*I + T*)*.* Both species showed significantly decreased transcript expression of *Myc*, and increased expression of *Il4r*, *Il13ra1*, *Il10ra*, and *Tgfbr2*. However, there was greater induction of *IL4r*, *IL10ra*, and *IL13ra1* in mouse; and *Tgfbr2* in rat. More prominent species differences were that in rat, *Il10rb* increased, and *Tgfb1* and *Pparg* decreased, while in mouse *Il1rn* and *Tgfbr1* increased. There were other apparent changes in “anti-inflammatory” genes that did not reach significance, likely because of the small sample size. These included reduced expression of *Mrc1* in both species, increased *Il1rn* (3.3-fold), *Arg1* (3.9-fold), and *Ccl22* (2.8-fold) in rat, and increased *Cd163* (2.9-fold) and *Ccl22* (21.3-fold) in mouse. [A pilot study of rat microglia at 6 h after I + T showed increases in Ccl22 and Arg1 (data not shown).] *M*(*IL-4*)*.* Both species showed increased expression of *Mrc1* and *Myc*, but with 1.5-fold greater induction in rat. *Ccl22* induction was also greater in rat (220.8-fold increase) than mouse (128-fold). Conversely, the increase in *Arg1* was much greater in mouse (1005-fold) than rat (6.3-fold). Species-specific changes were that *Il10rb* decreased in rat only, *Il10ra* and *Tgfbr2* decreased in mouse only; *Il1rn*, *Retnla*, *Pparg*, and *Tgfb1* increased in mouse only, and *Cd163* increased in rat only. [A pilot study of rat microglia at 6 h after IL-4 treatment found increases in Tgfb1, Mrc1, Arg1, Myc, Il4r, Il13ra1, and Cd163 (not shown).]

**Table 4:** Transcript expression of anti-inflammatory genes and receptors

	Control		I + T		IL-4		IL-10	
	Relative RNA counts ± SD		Fold change with respect to Control					
	Rat	Mouse	Rat	Mouse	Rat	Mouse	Rat	Mouse
*Arg1*	8 ± 4	20 ± 19	3.88	0.60	6.30	**1004.57** ^↑↑↑ ***^	0.93	6.18
*Ccl22*	9 ± 6	9 ± 4	2.83	21.34	220.81 ^↑↑↑^	128.20	0.81	0.64
*Cd163*	8 ± 4	3 ± 3	1.36	2.85	**30.08** ^↑↑↑ ***^	1.41	0.57	2.87
*IL1rn* (IL-RA)	4089 ± 1618	484 ± 206	3.25	**9.11** ^↑↑↑ ***^	0.23	**4.69** ^↑↑ ***^	3.48	0.98
*Il4*	10 ± 4	58 ± 8	0.90	0.53	0.56	0.77	1.04	0.99
*Il4r* (IL-4RA)	619 ± 34	147 ± 54	6.56 ^↑↑↑^	**12.08** ^↑↑↑ ***^	1.01	0.97	1.55	**12.24** ^↑↑↑ ***^
*Il10*	13 ± 12	22 ± 2	0.24	0.42	0.20	0.83	1.09	1.39
*Il10ra*	931 ± 101	408 ± 79	3.63 ^↑↑↑^	**6.74** ^↑↑↑ ***^	1.10	**0.44** ^↓ **^	1.09	1.09
*Il10rb*	1685 ± 135	2208 ± 227	**1.79** ^↑↑↑ ***^	1.16	**0.66** ^↓↓ ***^	1.16	**1.41** ^↑↑↑ *^	1.10
*Il13ra1*	563 ± 68	374 ± 65	2.35 ^↑↑↑^	**6.18** ^↑↑↑ ***^	0.73	0.52	1.33	**2.03** ^↑↑↑ *^
*Mrc1* (CD206)	1954 ± 959	1611 ± 864	0.03	0.09	**4.77** ^↑↑↑ **^	3.15 ^↑↑↑^	1.44	**3.00** ^↑↑ *^
*Myc*	676 ± 90	444 ± 79	0.22 ^↓↓↓^	0.15 ^↓↓↓^	**3.23** ^↑↑↑ ***^	2.11 ^↑↑↑^	1.10	**0.61** ^**^
*Pparg*	872 ± 379	101 ± 15	0.05 ^↓↓^	0.54	0.89	**2.57** ^↑↑↑ ***^	0.89	0.85
*Retnla* (FIZZ1)	6 ± 4	15 ± 6	1.26	1.31	1.15	**1036.01** ^↑↑↑ ***^	1.11	4.70
*Tgfb1*	17,115 ± 1112	2685 ± 234	**0.46** ^↓↓↓ ***^	0.99	1.16	1.28 ^↑↑↑^	1.14	0.91
*Tgfbr1*	3976 ± 654	1047 ± 193	1.11	**1.67** ^↑↑↑ ***^	0.72	0.68	1.14	0.72
*Tgfbr2*	1227 ± 105	1173 ± 191	**1.84** ^↑↑↑ *^	1.54 ^↑↑↑^	0.85	**0.44** ^↓↓↓ **^	1.35 ^↑↑^	1.57 ^↑↑↑^

Treatments, data presentation and analysis are as in Table 3

*M*(*IL-10*)*.* Responses were very different from IL-4. In both species, there was increased expression of *Tgfbr2*, but there were several species differences. In mouse, *Il4r*, *Il13ra1*, and *Mrc1* increased, while the only rat-specific change was an increase in *Il10rb*. There was a trend toward a decrease in *Myc* expression in mouse cells (*p* = 0.06).

**Fig. 4:**
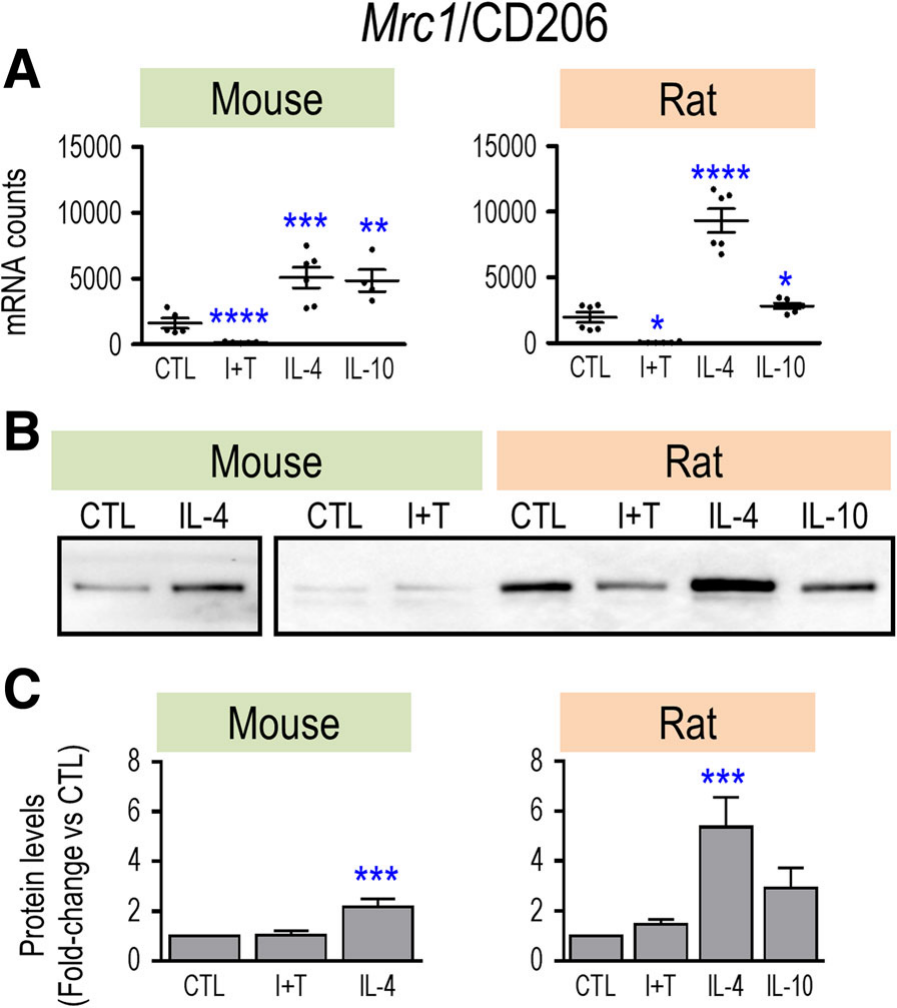
Species comparison of *Mrc1* mRNA and CD206 protein. Treatments and data presentation are as in Fig. 1. **a** *Mrc1* mRNA expression (mRNA counts/200 ng total RNA) was determined by NanoString analysis (mean ± SEM; *n* = 4–6 individual cultures), and plotted on the same *Y*-axis scale. **b** Representative Western blots of CD206 protein. **c** Summary of fold changes in CD206 protein expression (mean ± SEM; *n* = 4–6 individual cultures for mouse and 22 for rat), normalized to the total protein in each lane, and then to control microglia as in Fig. 1. Significant differences from unstimulated (control) cells are indicated: *p < 0.5; **p < 0.01; ***p < 0.001; ****p < 0.0001

**Fig. 5:**
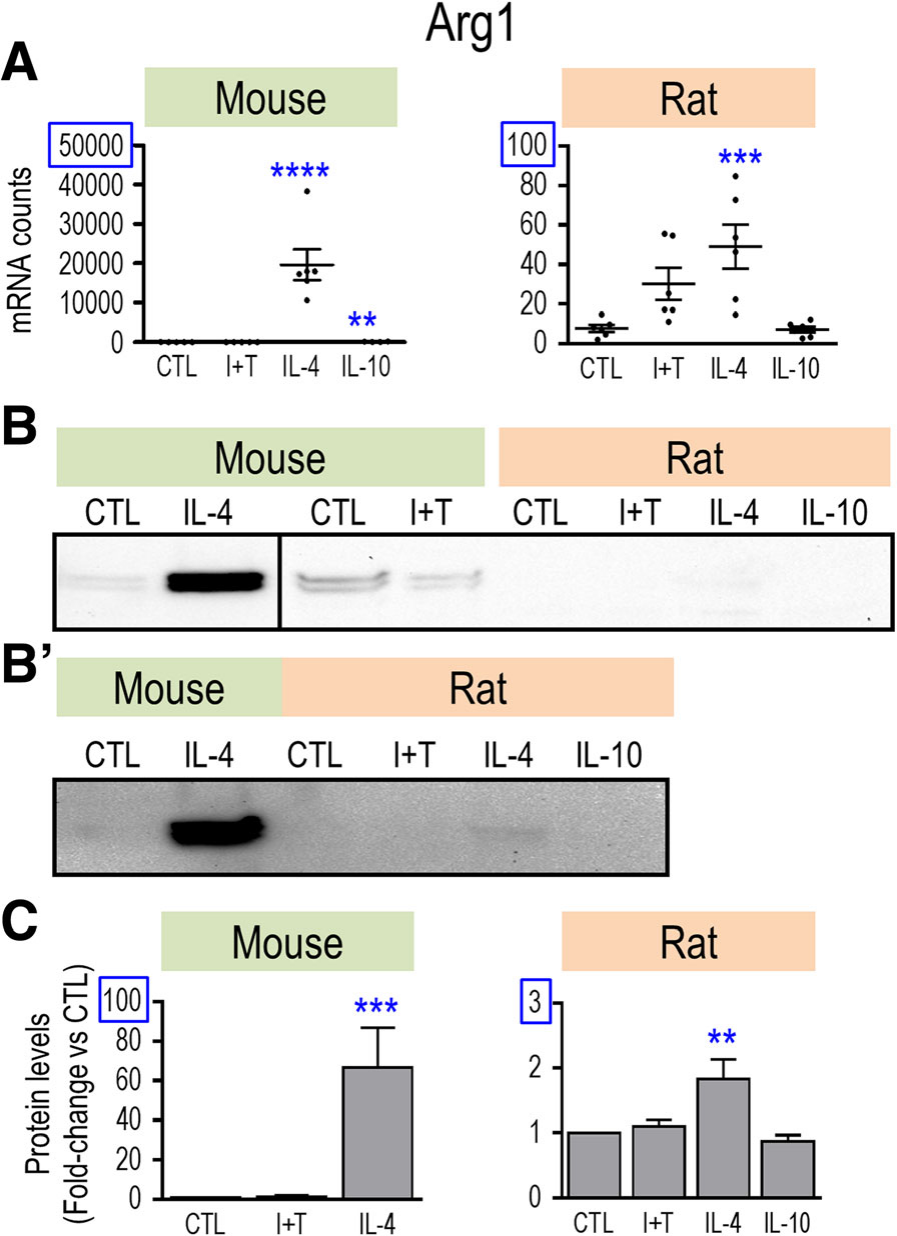
Species comparison of *Arg1* mRNA and protein. Treatments and data presentation are as in Fig. 1. **a** *Arg1* mRNA expression (mRNA counts/200 ng total RNA) was determined by NanoString analysis (mean ± SEM; *n* = 4–6 individual cultures). NB: The *Y*-axes differ (blue boxes placed for clarity). **b** Representative Western blots of Arg1 protein. **b’.** For clarity, to show detection of ARG1 in IL-4-treated rat microglia, an over-exposed blot is shown but was not used for quantification. **c** Summary of fold changes in Arg1 protein expression (mean ± SEM; *n* = 4–6 individual cultures for mouse and 21 for rat), normalized to the total protein in each lane, and then to control microglia as in Fig. 1. Again, note that the *Y*-axis for rat cells is much lower. Significant differences from unstimulated (control) cells are indicated: **p < 0.01; ***p < 0.001; ****p < 0.0001

Overall, the observed changes raise some concerns about whether genes that have been commonly used to indicate “alternative activation” (M2a) are, in fact, good markers for both rodent species. For instance, IL-4 treatment showed several species differences, and the M1 stimulus (I + T) increased *Arg1* in rat only and *Ccl22* in mouse only. Therefore, we examined protein changes for some key anti-inflammatory markers. As expected, CD206/MRC1 was induced by IL-4 treatment in both species and at both the mRNA (Fig. 4a) and protein levels (Fig. 4b, c). However, rat microglia showed higher CD206 protein levels in both resting and activated states (Fig. 4b), and a higher induction by IL-4 (5.4-fold vs 2.2-fold in mouse). For ARG1, the much lower mRNA counts in rat microglia (Fig. 5a) corresponded with much lower protein levels (Fig. 5b). As expected, IL-4 treatment increased both ARG1 mRNA and protein but the protein induction (Fig. 5b, c) was much higher in mouse cells (66.7-fold vs 1.8-fold). Thus, three observations suggest that ARG1 is a poor M2 marker in rat microglia: the very low mRNA and protein expression levels, low IL-4-mediated induction, and the unexpected mRNA increase seen with I + T.

### Microglia markers, immune modulators

**Fig. 6:**
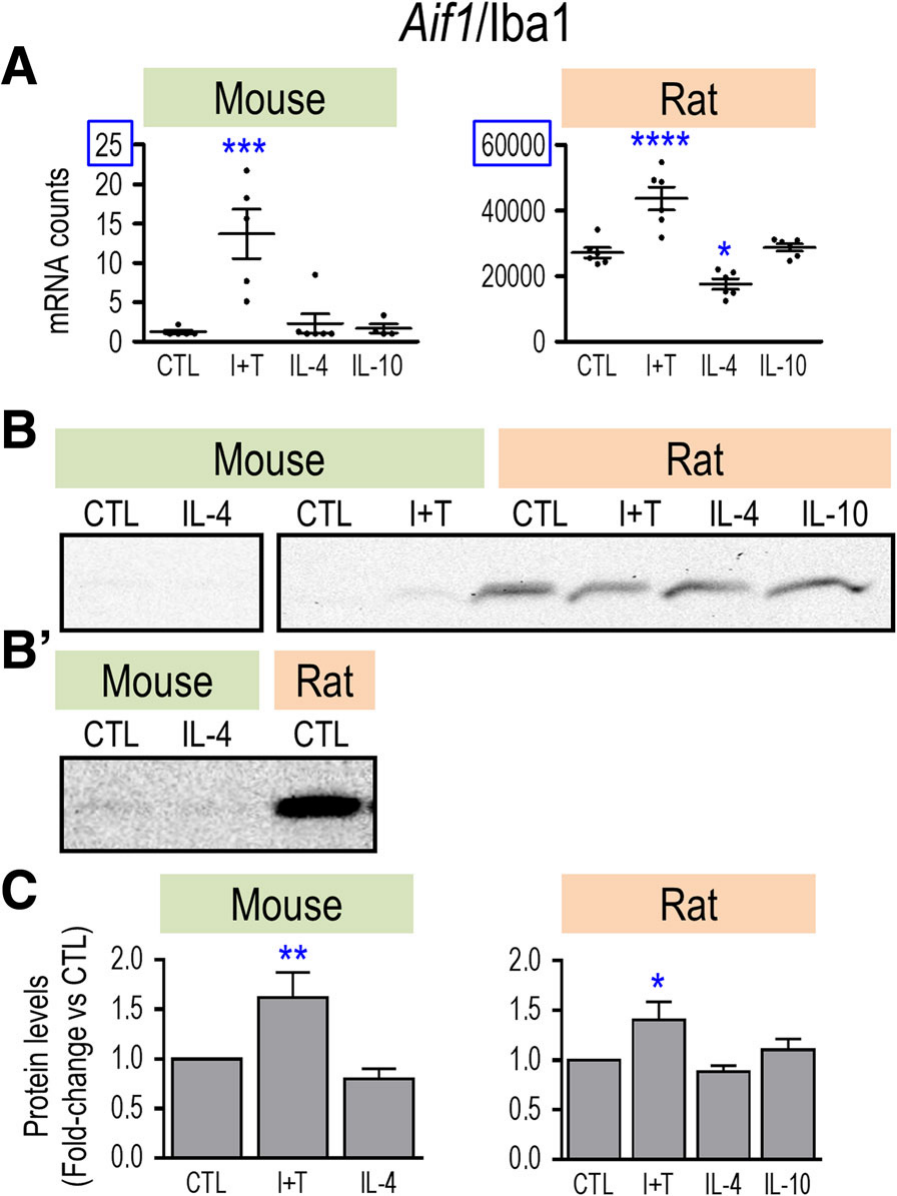
Species comparison of *Aif1* mRNA and Iba1 protein. Treatments and data presentation are as in Fig. 1. **a** *Aif1* mRNA expression (mRNA counts/200 ng total RNA) was determined by NanoString analysis (mean ± SEM; *n* = 4–6 individual cultures). NB: The *Y*-axes differ (blue boxes placed for clarity). **b** Representative Western blots of Iba1 protein. **b’** For clarity, to show detection of Iba1 in mouse microglia, an over-exposed blot is shown but was not used for quantification. **c** Summary of fold changes in Iba1 protein expression (mean ± SEM; *n* = 3–4 individual cultures for mouse and 14 for rat), normalized to the total protein in each lane, and then to control microglia as in Fig. 1. Here, the *Y*-axes are the same. Significant differences from unstimulated (control) cells are indicated: *p < 0.5; **p < 0.01; ***p < 0.001; ****p < 0.0001

We next examined several molecules routinely used to identify “activated” microglia after acute brain injury, and several immunomodulatory molecules. *Unstimulated.* Microglia of both species expressed low transcript levels of *Socs1* (suppressor of cytokine signaling 1) and *Socs*3 (Table 5; Additional file 4: Fig. S4). Both species showed moderate to very high levels of the other molecules examined: *Itgam* (CD11b), *Cd68*, *Cx3cr1*, toll-like receptor 2 (*Tlr2*), *Tlr4*, *Nfkbia* (IκBα; endogenous inhibitor of NFκB), *Tspo* (translocator protein), *Nr3c1* (glucocorticoid receptor, GR), and *Ptpn6* (Src homology region 2 domain-containing phosphatase-1; SHP-1). Interestingly, most control transcript levels were higher in rat than in mouse microglia, except for *Cx3cr1*, which was higher in mouse. Surprisingly, mRNA for *Aif1* (which codes for ionized Ca^2+^ binding adapter molecule 1, Iba1) was highly expressed in rat cells (>20,000 mRNA counts) but very low in mouse. This species difference was confirmed at the protein level, where Iba1 was much higher in unstimulated rat microglia (Fig. 6). *M*(*I + T*)*.* Both species showed increased transcript expression of *Nr3c1*, *Nfkbia*, *Socs1*, and *Tspo*, and decreased *Cd68* and *Cx3cr1*. The main species differences included the higher increase in mouse cells for *Socs1* (5.7-fold) and *Tspo* (4-fold), and the mouse-specific decrease in *Itgam* and increases in *Aif1*, *Ptpn6*, *Socs3* and *Tlr4* (which decreased in rat). Although both species showed modest increases in Iba1 protein (1.6-fold in mouse, 1.4-fold in rat), they did not reach statistical significance (Fig. 6c). *M*(*IL-4*)*.* Both species showed increased expression of *Tlr4* and decreased *Tlr2*. [A pilot study of rat microglia also showed an increase in Tlr4 at 6 h (not shown)]. *Socs1* increased in rat, and despite a 33-fold increase in mouse, it did not reach significance (*p* = 0.08). Notable species differences were increased *Cx3cr1* in rat but a dramatic decrease in mouse, decreased *Cd68* in rat but an increase in mouse, lower *Itgam* in rat, and lower *Ptpn6* in mouse. *M*(*IL-10*)*.* Both species showed increased expression of *Itgam* and *Tlr4*, and decreased *Cx3cr1*. Species differences were increases in *Cd68* and *Tspo* (rat only), and increase in *Nc3r1* (mouse only). Mouse had increased *Socs3*, but the 9.4-fold increase in rat was not significant. Interestingly, *Itgam* was the only gene selectively upregulated by IL-10 treatment in both species.

**Table 5:** Transcript expression of selected microglia markers and immune modulators

	Control		I + T		IL-4		IL-10	
	Relative RNA counts ± SD		Fold change with respect to Control					
	Rat	Mouse	Rat	Mouse	Rat	Mouse	Rat	Mouse
*Aif* (Iba1)	27,167 ± 3842	1 ± 1	1.61	**11.07** ^↑↑↑ ***^	0.65	1.85	1.06	1.35
*Cd68*	50,573 ± 302	8341 ± 540	0.55 ^↓↓↓^	0.63 ^↓↓↓^	**0.66** ^↓↓↓ ***^	1.24 ^↑^	**1.26** ^↑ **^	0.95
*Cx3cr1*	1073 ± 235	2819 ± 676	0.03 ^↓↓↓^	0.02 ^↓↓↓^	1.45 ^↑↑↑^	**0.05** ^↓↓↓ ***^	0.65 ^↓↓^	0.60 ^↓↓^
*Itgam* (CD11b)	6778 ± 1635	2767 ± 531	1.08	**0.37** ^↓↓↓ ***^	**0.67** ^**^	1.06	1.54 ^↑↑^	1.82 ^↑↑↑^
*Nfkbia* (IκBα)	5791 ± 2491	1057 ± 240	**5.77** ^↑↑↑ **^	4.73 ^↑↑↑^	0.57	0.58	0.95	1.08
*Nr3c1* (GR)	1540 ± 162	414 ± 37	**3.53** ^↑↑↑ **^	3.02 ^↑↑↑^	0.90	1.05	1.04	**1.42** ^↑ *^
*Ptpn6* (SHP-1)	2593 ± 401	2386 ± 318	1.11	**3.90** ^↑↑↑ ***^	1.1	**0.63** ^**^	1.15	1.07
*Socs1*	20 ± 7	10 ± 7	100.93 ^↑↑↑^	**576.90** ^↑↑↑ ***^	52.38 ^↑↑↑^	33.03	0.73	1.64
*Socs3*	62 ± 35	8 ± 5	10.69	**72.22** ^↑↑↑ ***^	1.07	1.41	9.44	**63.51** ^↑↑↑ ***^
*Tlr2*	3459 ± 1334	326 ± 104	1.22	1.61	0.28 ^↓^	0.14 ^↓↓^	1.37	0.84
*Tlr4*	720 ± 113	634 ± 46	0.58 ^↓^	**1.67** ^↑↑ ***^	**2.18** ^↑↑↑ ***^	1.45 ^↑^	1.49 ^↑^	1.58 ^↑^
*Tspo*	2289 ± 1053	655 ± 140	2.82 ^↑↑^	**11.22** ^↑↑↑ ***^	1.21	1.32	3.43 ^↑↑↑^	1.99

Treatments, data presentation and analysis are as in Table 3

Overall, the pro-inflammatory state in both species was marked by increased mRNA expression of *Nfkbia*. Several genes increased with I + T but were not selective pro-inflammatory markers. I + T increased *Nc3r1* and *Socs3* but they were also slightly increased with IL-10 in mouse; *Socs1* and *Tspo* were elevated in both species but also increased by IL-4 or IL-10 in rat. Several genes were not selective activation state markers in either species at the mRNA level: *Aif1*, *Itgam*, *Cd68*, *Cx3cr1*, *Tlr2*, *Tlr4*, and *Ptpn6*. Of note, I + T treatment decreased *Cx3cr1*, which is often used to identify microglia, and *Cd68*, which is often used to identify their phagocytic state.

### Purinergic and phagocytic receptors, and NOX enzymes

We conducted a species comparison of transcript levels of several molecules to follow up on our recent study of myelin phagocytosis and consequent production of reactive oxygen species (ROS) in rat microglia [17]. We also examined several purinergic receptors that can modulate microglial phagocytosis, ROS production, cytokine secretion, and migration [58]. *Unstimulated.* Microglia from both species had modest transcript levels (<500 mRNA counts/200 ng RNA) of *Nox1*, *Nox4*, *P2rx7*, *P2ry2*, and *P2ry12*; and higher levels (>2500 mRNA counts) of *Cybb* (NOX2), *Fcgr2b* (CD32), *Fcgr3a* (CD16), and *Msr1* (SR-A) (Table 6; Additional file 5: Fig. S5). The main species differences were the higher control levels in rat of *Fcgr1a* (CD64; 4.9-fold), *Ncf1* (9.3-fold), *Trem2* (31.2-fold), and *P2ry12* (3.4-fold). *M*(*I + T*)*.* Both species had decreased transcript levels of *Trem2*, and increased *Ncf1* (although 1.4-fold greater in rat), *Cybb* (1.8-fold greater in mouse), and *Fcgr3a* (1.5-fold greater in rat). Other species differences were the opposite changes in *Fcgr1a* expression, the increase in *P2ry2* in rat only, and decreases in *Msr1* and *P2ry12* in rat only. *M*(*IL-4*)*.* Both species showed decreased expression of *Cybb*. All other changes were species dependent, with most genes altered in rat cells but few in mouse. In rat, *Fcgr2b*, *P2rx7*, and *P2ry12* increased, while *Trem2* and *Msr1* decreased. Mouse-specific changes were decreased *P2ry12* and increased *Nox1*, which were opposite to the trends in rat. *M*(*IL-10*)***.*** In both species, *Fcgr2b* and *Fcgr3a* increased. Species differences were increases in *P2ry2* and *Trem2* in rat only, increased *Msr1* and *P2ry12* in mouse only, and opposite changes in *Cybb* (up in rat, down in mouse). *Nox4* transcripts remained low in both species and were unaffected by any treatment tested.

**Table 6:** mRNA expression of phagocytic and purinergic receptors and NOX enzymes

	Control		I + T		IL-4		IL-10	
	Relative RNA counts ± SD		Fold change with respect to Control					
	Rat	Mouse	Rat	Mouse	Rat	Mouse	Rat	Mouse
*Cybb* (NOX2)	3562 ± 601	7612 ± 1931	1.90 ^↑↑↑^	**3.48** ^↑↑↑ ***^	0.53 ^↓^	0.44 ^↓^	**1.45** ^**^	0.69
*Fcgr1a* (CD64)	6419 ± 2158	1301 ± 184	0.40	**6.59** ^↑↑↑ ***^	0.51	0.47	1.48	2.38
*Fcgr2b* (CD32)	5831 ± 1349	2995 ± 527	0.72	0.76	**4.01** ^↑↑↑ ***^	0.98	3.57 ^↑↑↑^	3.93 ^↑↑↑^
*Fcgr3a* (CD16)	8307 ± 4638	3778 ± 968	**3.77** ^↑↑↑ ***^	2.44 ^↑↑↑^	0.32	1.01	2.12 ^↑↑↑^	1.92 ^↑^
*Msr1* (SR-A/CD204)	7024 ± 1385	3196 ± 511	**0.10** ^↓↓↓ ***^	0.92	**0.30** ^↓↓↓ **^	0.78	1.27	**1.93** ^↑↑↑ ***^
*Ncf1*	8535 ± 2299	920 ± 80	**6.03** ^↑↑↑ ***^	4.17 ^↑↑↑^	0.58	0.99	0.94	1.37
*Nox1*	14 ± 6	10 ± 3	0.46	0.71	0.39	**4.90** ^↑↑↑ ***^	0.95	0.63
*Nox4*	2 ± 1	5 ± 3	3.26	0.66	1.28	0.90	1.76	0.43
*P2rx7*	190 ± 47	217 ± 30	1.31	0.86	**1.78** ^↑ **^	0.87	1.51	1.15
*P2ry2*	71 ± 14	57 ± 16	**4.22** ^↑↑↑ ***^	1.12	2.08	1.35	2.57 ^↑↑^	1.30
*P2ry12*	449 ± 52	132 ± 60	**0.35** ^↓↓ *^	0.87	**1.82** ^↑↑↑ ***^	0.39 ^↓↓^	0.95	**1.53** ^↑ *^
*Trem2*	6515 ± 982	204 ± 11	0.04 ^↓↓↓^	0.12 ^↓↓↓^	**0.48** ^↓↓↓ ***^	1.09	**1.25** ^↑ ***^	0.79

Treatments, data presentation, and analysis were as in Table 3

### Expression of Kir2 and Kv1 channel genes

**Fig. 7:**
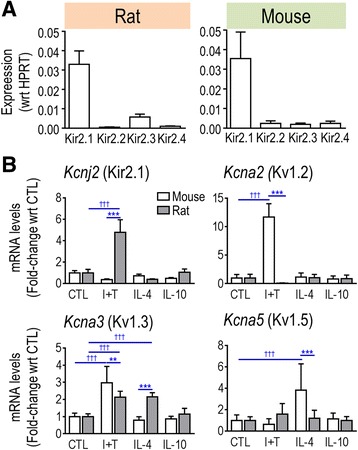
K^+^ channel transcript expression rat and mouse microglia were unstimulated (CTL) or stimulated with IFN-γ and TNF-α (I + T), IL-4 or IL-10. **a** Real-time qRT-PCR analysis of the expression of Kir2 subfamily members in unstimulated rat and mouse microglia (*n* = 6 individual cultures). **b** mRNA expression (mRNA counts/200 ng total RNA) was determined by NanoString and expressed as fold change relative to unstimulated control cells (mean ± SEM; *n* = 4–6 individual cultures). **p < 0.01; ***p < 0.001

Primary rat and mouse microglia express Kir2.1 mRNA and protein [18, 24] but other Kir2-family members have not been assessed. Because these channels can function as homotetramers or heterotetramers [59], we first compared expression of *Kcnj2* (Kir2.1), *Kcnj12* (Kir2.2), *Kcnj4* (Kir2.3), and *Kcnj14* (Kir2.4). [We omitted Kir2.5 because it is electrically silent and Kir2.6 because it is expressed primarily in skeletal muscle [60, 61]]. In both rodent species, transcript expression of Kir2.1 (*Kcnj2*) predominated (Fig. 7a). In a pilot study, NanoString showed that expression of *Kcnj12*, *Kcnj4*, and *Kcnj14* in rat microglia was not changed in M(I + T), M(IL-4), or M(IL-10) states, and real-time RT-PCR corroborated these results (data not shown). Thus, homomeric Kir2.1 channels likely produce the inward-rectifying K^+^ current in both species, which is important because the blocker we used (ML133) can affect other Kir2 members [62].

Kv1.2 (*Kcna2*), Kv1.3 (*Kcna3*), and Kv1.5 (*Kcna5*) mRNA and protein have been detected in primary rat microglia [28, 29, 63, 64]. In primary mouse microglia, Kv1.3 and Kv1.5 have been detected [65], but we found no reports of Kv1.2 expression. Here, we compared transcript expression of *Kcna2*, *Kcna3*, and *Kcna5*, and *Kcnj2* (Kir2.1) in different activation states in both species (Fig. 7b). *Unstimulated* ***.*** The main species difference was the 5.2-fold higher Kir2.1 mRNA expression in rat microglia (1984 ± 656 (SD) mRNA counts in rat vs 382 ± 82 in mouse). Both species expressed relatively low transcript levels of Kv1.2 (77 ± 50 mRNA counts in rat vs 9 ± 5 in mouse), Kv1.3 (113 ± 19 mRNA counts in rat vs 63 ± 13 in mouse), and Kv1.5 (4 ± 2 mRNA counts in rat vs 3 ± 1 in mouse). *M*(*I + T*)***.*** Two channels showed opposite changes. Kir2.1 expression increased 4.8-fold in rat but decreased 2.8-fold in mouse; Kv1.2 decreased 10.8-fold in rat but increased 11.7-fold in mouse. Kv1.3 expression increased in both species but to a greater degree in mouse. Kv1.5 was unchanged in both species. *M*(*IL-4*)***.*** In mouse, the only effect was a 3.8-fold increase in Kv1.5 but the mRNA level remained very low (~10 counts/200 ng RNA). In rat cells, Kv1.3 increased 2.2-fold. [In a pilot study on rat cells, Kv1.3 mRNA also increased at 6 h (data not shown).] *M*(*IL-10*)***.*** There were no significant changes. Overall, the most notable species differences were the opposite changes evoked by I + T in Kir2.1 and Kv1.2, and the IL-4-evoked increase in Kv1.3 in rat only and Kv1.5 in mouse only.

The next step was to use electrophysiology to compare the currents under each activation state. This is a more accurate readout than simply measuring protein levels because ion channel function can be affected by post-translational modulation and trafficking to the surface membrane; for instance, as we have shown for Kv1.3 in microglia [28, 50]. Here, we measured total inward and outward currents, and then used specific voltage protocols and channel blockers to isolate and quantify Kir2.1 and Kv1.3 currents. Because microglial morphology changes with M1 activation [19, 20, 56] and potentially affects cell size, we first determined that the cell capacitance, which is proportional to cell size, did not differ under any activation condition or between species (Table 7). Subsequently, we recorded currents from microglia with the most prevalent morphologies; i.e., unipolar for unstimulated, M(IL-4) and M(IL-10) cells; rounded or amoeboid for M(I + T) cells.

**Table 7:** Cell capacitance of rat and mouse microglia in different activation states

		Capacitance (pF); mean ± SEM (*n*)		Rat versus mouse
	Morphology	Rat	Mouse	
Control	Unipolar	25.8 ± 1.2 (54)	24.1 ± 1.4 (46)	*ns*
I + T	Amoeboid	29.8 ± 2.4 (30)	25.0 ± 1.0 (45)	*ns*
IL-4	Unipolar	25.4 ± 1.4 (48)	24.8 ± 1.2 (29)	*ns*
IL-10	Unipolar	28.1 ± 1.3 (34)	29.0 ± 2.0 (27)	*ns*

Data are expressed as mean capacitance (pF) ± SEM (number of cells). Based on two-way ANOVA with Bonferroni’s post hoc test, there were no differences between species for any activation paradigm or between activation states within a species

### Inward-rectifier (Kir) current

**Fig. 8:**
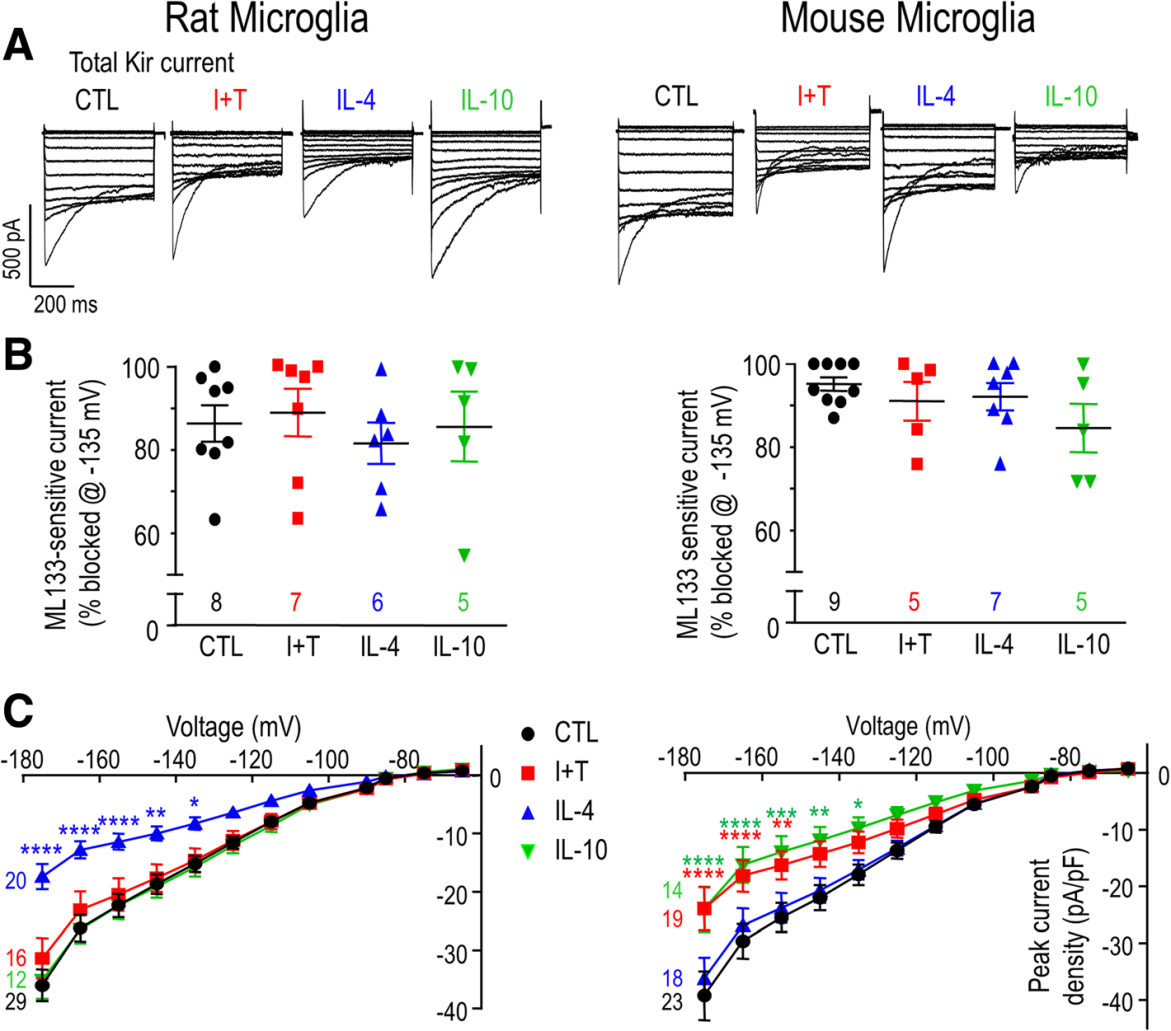
Inward-rectifier (Kir) current versus activation state. Rat and mouse microglia were unstimulated (CTL) or stimulated for 30 h with IFN-γ and TNF-α (I + T), IL-4 or IL-10. Whole-cell Kir currents were recorded in response to a voltage protocol with test pulses between −175 and −65 mV in 10-mV increments from a holding potential of −15 mV. **a** Representative traces of total Kir current in primary rat (left column) and mouse (right column) microglia. **b** Scatterplot of individual cells showing the proportion of the peak inward current (at −135 mV) that was blocked by 20 μM ML133. **c** Current-voltage (*I-V*) relations for the total Kir current, where peak current density (pA/pF) was plotted as a function of voltage. Data are shown as mean ± SEM (number of cells). *p < 0.5; **p < 0.01; ***p < 0.001; ****p < 0.0001

Microglia Kir currents displayed the stereotypical features of Kir2.1 (Fig. 8a). This current activates at negative potentials due to relief of channel block by internal Mg^2+^ and polyamines [59, 66], and then the current relaxes at very negative potentials due to time-dependent block by external Na^+^ [52, 67].

To quantify the Kir2.1 component of the whole-cell current, we used ML133, a membrane-permeant blocker [62] that acts in a time-dependent manner with an IC_50_ ~ 3.5 μM in rat microglia [18]. Regardless of the microglial activation state, most of the Kir current (82–86% in rat, 85–95% in mouse) was blocked by 20 μM ML133 (Fig. 8b), and the small remaining current had a linear current-versus-voltage (*I-V*) relation (not shown). Thus, to examine whether microglial activation states affect channel activity, we compared the total Kir current density. In both species, the *I-V* relations showed inward rectification and reversal at about − 82 mV after junction potential correction (Fig. 8c), which is close to the K^+^ Nernst potential. *Unstimulated.* Both rodent species displayed a similar magnitude of Kir2.1 current (Fig. 8a, c) despite the differences in mRNA counts (Fig. 7b). Species differences were seen under all activation states examined (Fig. 8c). *M*(*I + T*)*.* The Kir2.1 current decreased in mouse cells but was unchanged in rat. *M*(*IL-4*)***.*** The Kir2.1 current decreased substantially in rat cells but was unchanged in mouse. *M*(*IL-10*)***.*** The Kir2.1 current decreased in mouse cells but was unchanged in rat. Overall, the Kir2.1 current amplitude is not a reliable indicator of the microglial activation state, but instead depends on both the rodent species and activating stimulus.

### Outward-rectifier (Kv) current

#### Rat microglia

**Fig. 9:**
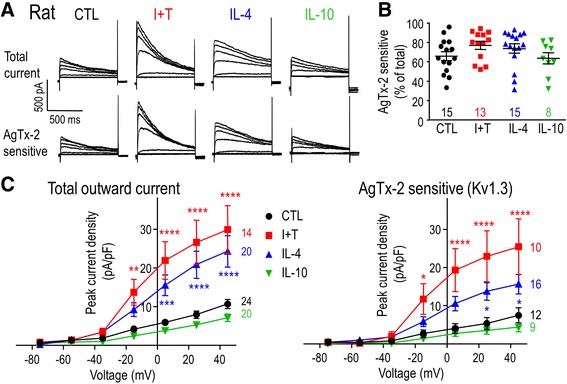
Rat microglia: AgTx-2 sensitive Kv1.3 currents versus activation state. Whole-cell Kv currents were isolated using a voltage clamp protocol holding at −105 mV, followed by 1-s-long voltage steps from −75 to +45 mV in 20-mV increments, applied every 60 s. **a** Representative traces of total Kv current in unstimulated (CTL) rat microglia, and in cells stimulated for 30 h with IFN-γ and TNF-α (I + T), IL-4 or IL-10. For each cell, 5 nM AgTx-2 was perfused into the bath to record the AgTx-2 insensitive component, which was then subtracted from the total current to yield Kv1.3 current. **b** Scatter plot of individual cells showing the proportion of the peak current (at +45 mV) that was blocked by AgTx-2. **c** Peak current density (pA/pF) as a function of voltages for the total Kv (left) and the AgTx-2-sensitive Kv1.3 (right) currents. Data are shown as mean ± SEM (number of cells). *p < 0.5; **p < 0.01; ***p < 0.001; ****p < 0.0001

Kv currents were observed in every rat microglial cell examined under all activation conditions (Fig. 9a), and the proportion blocked by AgTx-2 was similar (64–77%; Fig. 9b). The remaining current had a nearly linear *I-V* relation without time dependence during steps (not shown) and was not identified. In all activation states, the total Kv current and AgTx-2-sensitive Kv1.3 component activated in a time- and voltage-dependent manner above about − 60 mV (corrected for junction potential and leak), and increased with depolarization (Fig. 9a, c). Time-dependent inactivation was also apparent during depolarizing test pulses (Fig. 9a). M(I + T) and M(IL-4) both increased total Kv current and the AgTx-2-sensitive component (Fig. 9c). [In a pilot study, IL-4 increased the Kv current by more than twofold at 6 and 24 h (data not shown).] In contrast, M(IL-10) treatment did not affect the current amplitude.

#### Mouse microglia

**Fig. 10:**
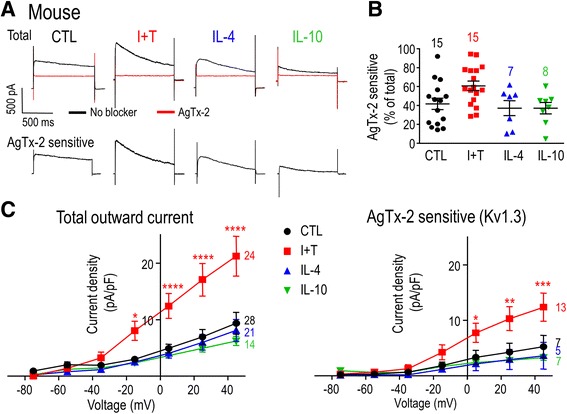
Mouse microglia: AgTx-2 sensitive Kv1.3 currents versus activation state. Whole-cell Kv currents were recorded using a modified voltage protocol. From a holding potential of −105 mV, a single step to +45 mV was applied for 1 s before returning to −105 mV for 60 s, and then a voltage ramp was applied from −75 to +45 mV over 120 ms. **a** Representative traces of Kv currents in the absence and presence of 5 nM AgTx-2 at +45 mV in unstimulated (CTL) cells, and in cells stimulated for 30 h with IFN-γ and TNF-α (I + T), IL-4, or IL-10. For each cell, AgTx-2 was perfused into the bath to record the AgTx-2-insensitive component, which was then subtracted from the total current to yield the Kv1.3 current. **b** Scatter plot of individual cells showing the proportion of the peak current (at +45 mV) that was blocked by AgTx-2. **c** Peak current density (pA/pF) as a function of voltage was plotted from the voltage-ramp component: total Kv current (left), AgTx-2 sensitive Kv1.3 current (right). All data are shown as mean ± SEM (number of cells). *p < 0.5; **p < 0.01; ***p < 0.001; ****p < 0.0001

Examples of Kv currents in mouse microglia under each activation state are shown in Fig. 10a. The first striking species difference was their lower prevalence in mouse; i.e., ~ 56% (19/34) of unstimulated cells, ~ 42% (10/24) of M(IL-4) cells, and ~ 56% (9/16) of M(IL-10) cells. Interestingly, ~ 92% of M(I + T) cells had Kv currents (23/25 cells). A second species difference was that in mouse cells expressing a Kv current, AgTx-2 blocked a lower proportion of the Kv current: ~ 42% (15 cells) in unstimulated cells, ~ 37% (7 cells) in M(IL-4) cells, ~ 37% (8 cells) in M(IL-10) cells, and ~ 61% (17 cells) in M(I + T) cells (Fig. 10b). This suggests that mouse microglia express at least one additional Kv current. The AgTx-2 sensitive Kv1.3 current in mouse microglia, similar to rat cells, activated at about −50 mV after junction potential correction (Fig. 10c). We then compared the total Kv and AgTx-2 sensitive current densities under the different activation states. M(I + T) cells had larger Kv and Kv1.3 currents (Fig. 10c), which was consistent with the mRNA data (Fig. 7b), and similar to rat microglia (Fig. 9). Unlike rat, M(IL-4) mouse cells had the same current densities as unstimulated cells. As for rat cells, M(IL-10) had no effect.

While changes in the prevalence and amplitude of Kv and Kv1.3 currents were seen with the stimuli tested, these currents are apparently not reliable indicators of the microglial activation state and also differ with the species.

### The activation state affects morphology, migration, and role of K^+^ channels

**Fig. 11:**
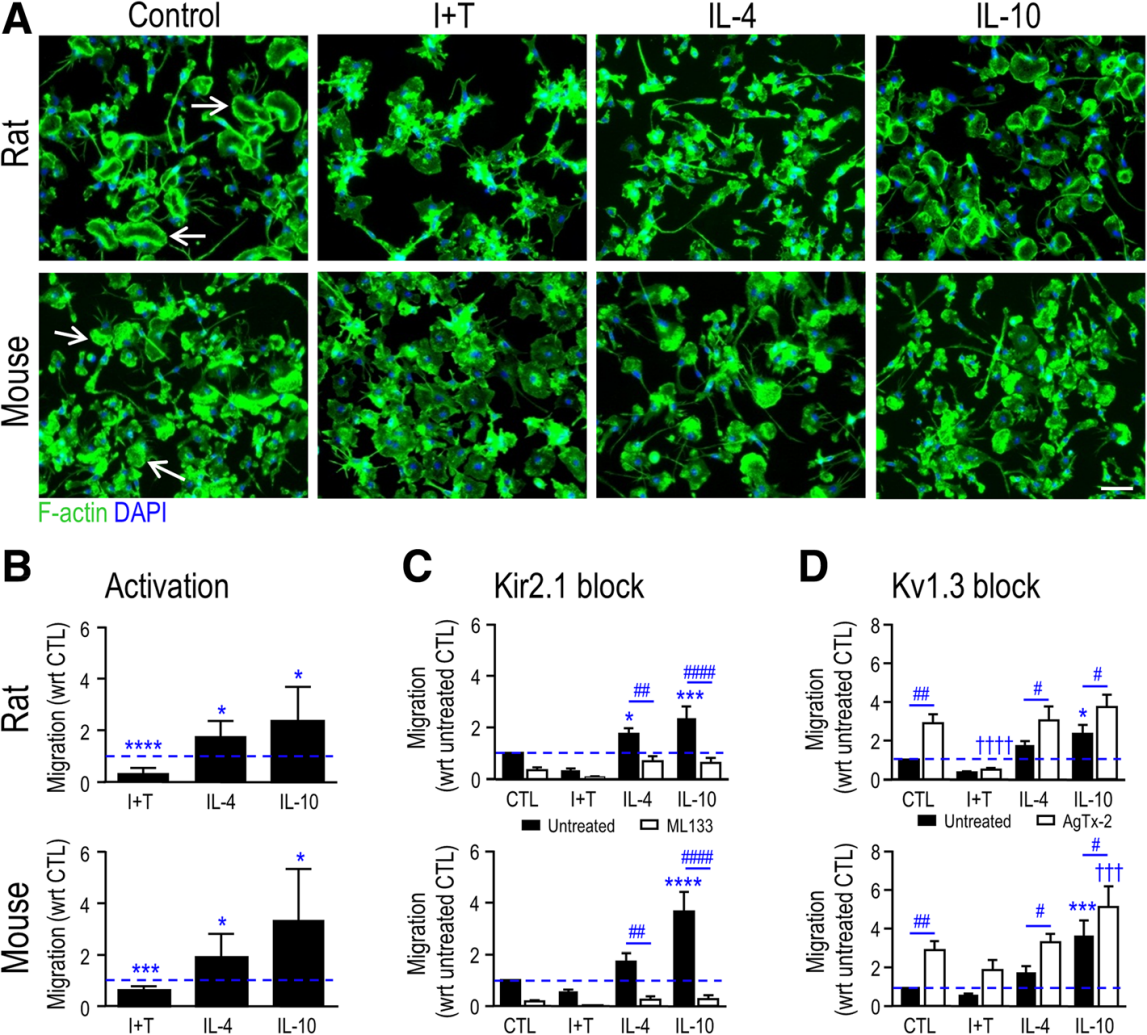
Kir2.1 and Kv1.3 activities contribute to microglial migration. **a** The activation state affects the migratory phenotype. Rat and mouse microglia were unstimulated (CTL) or stimulated with IFN-γ and TNF-α (I + T), IL-4, or IL-10 for 24 h. Representative images of neonatal rat and mouse primary microglia labeled with phalloidin to visualize F-actin (green) and DAPI to label nuclei (blue). Many unipolar microglia (except in I + T-treated cells) have a migratory phenotype with a single large lamellum that contains an F-actin-rich ring (a “podonut”; examples shown by arrows). Scale bar, 50 μm. **b** Microglia migration is affected by the activation state. All graphical results are expressed as fold change normalized to unstimulated cells (indicated by dashed lines). **c** Microglia migration with or without 20 μM ML133 to block Kir2.1 channels. **d** Microglia migration with or without 5 nM AgTx-2 to block Kv1.3 channels. Data are shown as mean ± SEM (*n* = 6–9 individual cultures) and were analyzed by one-way ANOVA with Dunnett’s post hoc test (activation state) or two-way ANOVA with Bonferroni’s post hoc test (when channel blockers were used). The comparisons are * differences between CTL and stimulated cells; † CTL versus activated cells treated with a channel blocker; # effects of a channel blocker within a given activation state. One symbol indicates *p* < 0.05, two symbols, *p* < 0.01, three symbols, *p* < 0.001, four symbols, *p* < 0.0001

Very little is known about migration of mouse microglia in different activation states or roles of Kir2.1 and Kv1.3 channels in migration in either rodent species (see “Discussion”). Therefore, we next compared these aspects under all four activation states, starting with morphology. We had previously shown that unipolar rat microglia migrate in the direction of the lamellum, which usually contains a prominent ring of F-actin-rich podosomes that we called a “podonut” [19, 31, 68]. Podosomes are subcellular structures involved in migration through roles in cell adhesion and matrix degradation [69]. *Unstimulated.* Both species showed a similar initial morphology. Many were unipolar, with a uropod and a large lamellum that often contained a podonut (Fig. 11a). There were both similarities and differences in their morphological responses to activating stimuli. Broadly speaking, unipolar microglia were common in highly migratory, unstimulated and M2 states, while round or amoeboid cells were common for M1 microglia of both species. *M*(*I + T*)*.* The shape of the cells changed dramatically, but with subtle species differences. Rat microglia were clustered into chains of cells, while mouse microglia were more evenly distributed. For both species, but especially in mouse microglia, individual cells often appeared flat and round or amoeboid. There was no apparent cell polarity but the cells often bore short, spiky processes. *M*(*IL-4*)***.*** Both species showed many unipolar cells, and many of them contained a podonut. The morphology of rat microglia was more heterogeneous and lamellae were often smaller than in unstimulated rat cells or M(IL-4) mouse cells. *M*(*IL-10*)***.*** For rat microglia, we previously reported that IL-10 increases the proportion of cells containing podonuts [20]; however, here, the mouse microglia continued to resemble unstimulated cells.

Next, we quantified migration in each activation state. *Rat.* M(I + T) cells migrated 2.9-fold less than unstimulated cells, while M(IL-4) and M(IL-10) migrated more, by 1.8-fold and 2.4-fold, respectively (Fig. 11b). Blocking Kir2.1 channels (20 μM ML133) reduced migration by 2.6-fold in M(IL-4) cells and 3.7-fold in M(IL-10) cells (Fig. 11c). Although ML133 reduced migration by 2.8-fold in unstimulated cells and 4-fold in M(I + T) cells, ANOVA did not show statistical significance, likely because migration was not as high as with IL-4 or IL-10 treatment. Blocking Kv1.3 (5 nM AgTx-2) increased migration of unstimulated microglia (2.9 fold), M(IL-4) cells (1.7 fold), and M(IL-10) cells (1.6 fold) (Fig. 11d). *Mouse.* Effects of the activation state on migration were similar to rat. Migration decreased 1.6-fold in M(I + T) cells, increased 1.9-fold in M(IL-4) cells, and 3.3-fold in M(IL-10) cells. Blocking Kir2.1 also inhibited migration of mouse microglia: by 7.2-fold in M(IL-4) cells and 14.2-fold in M(IL-10) cells. Again, while ML133 apparently reduced migration by 5.8-fold in unstimulated mouse microglia and by 20.8-fold in M(I + T) cells, ANOVA did not show statistical significance. Similar to rat cells, blocking Kv1.3 with AgTx-2 increased migration of unstimulated mouse microglia (2.9 fold), M(IL-4) cells (1.9 fold), and M(IL-10) cells (1.4 fold). While AgTx-2 appeared to increase migration of M(I + T) cells (3.2 fold), it was not statistically significant. For both species, we ruled out changes in the number of cells available to migrate. That is, a CyQuant NF assay showed that neither channel blocker altered the cell density over the 24-h migration period, regardless of activation state (data not shown).

Together, our results suggest that in microglia of both species, Kir2.1 activity facilitates migration and Kv1.3 activity inhibits it, regardless of the activation state.

## Discussion

We compared gene expression, six proteins, two ion currents, and the role of these channels under four defined activation states in primary rat and mouse microglia. We found both similarities and differences between these species. Because several activation paradigms and outcomes were examined; for clarity, the most relevant literature will be discussed under four topics: molecular profiling of activation responses; Kir2.1 channel expression and current; Kv1.3 channel expression and current; and contributions of Kir2.1 and Kv1.3 channels to migration. In addition, for ease of comparison with the literature, protein names will be used if the gene names are not commonly used (all gene names are in Tables 1 and 2). Finally, we will focus the discussion on primary microglia because cell lines can differ in their molecular profile, activation responses, and ion channel expression [32, 33, 35, 50, 70].

### Molecular profiling of activation responses of primary rat and mouse microglia

Transcriptomics is increasingly used to profile responses of cells or tissues (e.g., after damage or disease). The information obtained can stand on its own or be used to decide which genes are interesting candidates for further study, including protein analysis by immunohistochemistry or Western blots. Most DNA is transcribed [71], and thus, differences in mRNA generally produce differences in protein. Exceptions to this can occur if a protein is either unusually stable or unstable, or if specific miRNAs interfere with mRNA translation or lead to mRNA degradation. For the proteins we examined by Western analysis (iNOS, COX-2, PYK2, CD206, ARG1, Iba1), the results correlated well with the mRNA data and further support the observed species differences.

While most studies of microglial activation have used primary rat or mouse cells, very few direct species comparisons have been made and the cell activation state was often not determined. We found one older study that directly compared primary rat and mouse microglia but it was restricted to glutamate secretion from unstimulated cells [9]. An interesting recent in vitro study found differences between responses of rat, mouse, and human microglia to oxygen glucose deprivation (OGD) but the resulting activation state was not determined [8]. In that study, rat and human microglia were similar but mouse cells differed in cytokine mRNA expression (IL-1α, IL-1β, IL-6, IL-10, and TNF-α) both at baseline and after OGD. In contrast, mouse and human levels of several chemokines (CX3CL1, CXCL10, CXCL12, CCL2, CCL3) were unaffected by OGD, but increased in rat microglia. A small number of in vivo studies have directly compared inflammatory responses in rat and mouse CNS but the microglial activation state was not determined. For instance, after focal cerebral ischemia in mice, induction of pro-inflammatory mediators (IL-1β, iNOS, TNF-α) in the infarcted tissue was lower and more delayed than in rat [72]. In another study, following intracortical microelectrode implantation, CD68 immunoreactivity declined over several weeks in rat but not in mouse [73]. Following spinal cord contusion, rats and mice had similar microglia/macrophage accumulation in the lesion (maximal infiltration by 7 days); however, mice had delayed and/or protracted infiltration of T lymphocytes and dendritic cells, and a unique cellular response consisting of clusters of fibrocytes forming a clear fibrous scar [74].

#### *Unstimulated (control) microglia*

Importantly, we first used numerous gene markers to validate that their starting (control, unstimulated) state was similar. For instance, as expected for “resting” microglia, both species showed very low mRNA levels of numerous pro-inflammatory mediators (iNOS, IL-6, COX-2, IFN-γ, IL1R1) and anti-inflammatory mediators (ARG1, CCL22, FIZZ1, CD163, IL-4, IL-10). Both species also had similar (moderate to high) mRNA levels of several cytokine receptors (TNFR1, TNFR2, IFNGR1, TGFBR1, TGFBR2, IL-RA, IL-4Rα, IL-10RA, IL-10RB, IL-13Rα1) as well as ICE, MRC1, MYC, PYK2, TGF-β1 and TNF-α.

*M*(*I + T*). This pro-inflammatory stimulus evoked several similar gene responses in rat and mouse microglia, although there were quantitative differences. There were increased transcript levels of several well-known pro-inflammatory genes (iNOS, IL-6, TNF-α), NOX enzymes (NOX2, NCF1), and some less frequently examined genes (PYK2). Previously, increased PYK2 immunoreactivity was seen in rat microglia in vivo after transient middle cerebral artery occlusion or kainate-induced seizures [75], and although their activation state was not determined, our results suggest they were in a pro-inflammatory state. Several results are useful when considering the possible complexity of microglial responses to additional or sustained stimuli. The two species showed opposite changes in transcript expression of the innate immune receptor, TLR4, suggesting that they would respond differently to further incoming pro-inflammatory signals. In both species, I + T treatment increased transcript levels of receptors and immunomodulators that promote anti-inflammatory or deactivating signaling cascades and inflammation resolution. We recently found that M(I + T) rat microglia responded to subsequent IL-4 exposure with dampened pro-inflammatory responses [17]. Similarly, in M(LPS) mouse microglia, the pro-inflammatory responses were dampened by IL-4 treatment [76].

*M*(*IL-4*). IL-4 evoked several important species differences in gene expression. We found that two markers commonly used to identify IL-4-mediated alternative activation in mice, FIZZ1 and PPAR-γ [14, 77], were not induced in microglia from Sprague-Dawley rats. CD163 is considered a marker of anti-inflammatory microglia [14], and a marker of perivascular macrophages in the unperturbed brain [78]. We found that IL-4 treatment increased CD163 transcripts in rat microglia but, in mouse, none of the cytokines tested increased it. MRC1 is often considered an alternative-activation marker. Although it was selectively induced by IL-4 in rat microglia, it was also induced by IL-10 in mouse, as previously reported [76]. Thus, molecules used to identify alternative activation in one rodent species are not always generalizable.

*M*(*IL-10*). In both rodent species, responses to IL-10 often differed from IL-4. For instance, IL-10 did not reduce transcript expression of pro-inflammatory mediators (NOX2, IL-1β, IFNGR1) or increase anti-inflammatory mediators (CCL22, MYC, ARG1). Opposite effects were sometimes seen in the two species (FcγRIa, IL-13Rα1, TGFBR2). Surprisingly, M(IL-10) and M(I + T) microglia shared many similarities, but not in induction of pro-inflammatory mediators. Instead, both stimuli induced transcripts of molecules known to modulate inflammatory responses (FcγRIIIa, IL-4Rα, IL-13Rα1, SOCS3, TGFBR2, TSPO), but the IL-10 responses were either comparable or lower than I + T responses. Induction of these modulatory genes suggests that self-limiting feedback responses occurred. Similarly, an earlier study of mouse microglia showed that IL-10 increased IL-1RN and SOCS3 mRNA levels and production of IL-6, CXCL1 and CCL2, but generally to a lesser extent than stimulation with LPS, TNF-α, or IL-1β [76].

There are important implications of these molecular profiles of microglial inflammatory responses. (i) Some molecules routinely used to identify “activated” microglia/macrophages in vivo (CD11b, CD68) were not selectively increased by the pro-inflammatory stimulus, I + T. However, Iba1 was selectively increased in both species. (ii) The only genes induced selectively by IL-10 were CD11b (rat and mouse), CD68, and TREM2 (rat only), and FcγRIIb, P2Y12, and SR-A (mouse only). CD11b and CD68 are often used as markers of microglial “activation” in vivo [79, 80], but they differ in temporal and spatial patterns after ischemia in the mouse cortex [81]. Because CD11b and CD68 are receptors involved in phagocytosis by microglia, they are expected to be important after CNS damage [82, 83]; thus, it is surprising that neither was elevated by I + T (or IL-4) in either species. (iii) CX_3_CR1 expression was surprisingly malleable and species dependent. This receptor is restricted to microglia in the CNS, where it binds neuron-derived CX_3_CL1, and helps maintain microglial quiescence [84]. While the CX_3_CR1 transcript levels increased slightly after IL-4 treatment only in rat microglia, the most striking change was a decrease following all cytokine treatments in mouse. It is difficult to predict the outcome of these changes. CX_3_CR1-null mice are said to display microglial hyper-activation [84] but its role is apparently damage-dependent because CX_3_CR1 deletion was beneficial in models of transient ischemia [85] and Alzheimer’s [86] but harmful in Parkinson’s and ALS models [87].

### Changes in Kir2.1 expression and current in activated rodent microglia

Previously reported changes in Kir and Kv currents with microglial activation have been inconsistent and hinted at species differences.

The first reports of Kir2.1 mRNA transcripts in microglia were in the mouse cell lines, BV-2 and C8-B4 [25, 88], and their activation state was not addressed. Recently, we showed that primary rat microglia express Kir2.1 mRNA transcripts [18]. The present study is apparently the first to show that microglia from both species express transcripts for several members of the Kir2 family (Kir2.1, Kir2.2, Kir2.3, Kir2.4) and that Kir2.1 transcript expression was much higher and was the only subtype that responded to the activating stimuli.

While the mRNA results suggest that the Kir current in rodent microglia is produced by Kir2.1, numerous patch-clamp studies have not confirmed its identity. It is useful to compare electrophysiological and pharmacological properties of the microglial current with cloned Kir2.1 channels. Like cloned Kir2.1, the microglial current shows fast activation kinetics, Na^+^-dependent relaxation at very hyperpolarized potentials, a single channel conductance of 25–30 pS [25, 52, 89], and a small outward current above the reversal potential [18, 90]. There is no selective inhibitor of Kir2.1 channels, and most studies have used the pan-Kir blocker, Ba^2+^. It is important to note that Ba^2+^ is a poor blocker at depolarized potentials [18], and this will compromise its value in cell function assays that address roles of Kir channels. A better blocker is the Kir2 family-specific blocker, ML133 (20 μM), which is not voltage dependent and blocks most of the Kir current in primary rat microglia within several minutes [18]. In the present study, a similar block by 20 μM ML133 was seen in rat (86%) and mouse (95%) microglia. Because ML133 takes time to enter the cell and act on an internal site on the channel [62], its efficacy depends on the duration of drug exposure. For mouse microglia, it was recently reported that 20 μM ML133 blocked only ~ 48% of the Kir current [91] but the exposure time was not stated.

Published responses of the Kir current to microglial activating stimuli have been quite variable. While the Kir current is present in most unstimulated rat and mouse microglia, effects of pro-inflammatory stimuli in rat microglia range from no change to a small increase (with LPS) or a large increase (with IFN-γ) [27, 52, 92, 93]. In contrast, in primary mouse microglia, LPS or IFN-γ reduced the Kir2.1 current [27, 94, 95]. Our direct species comparison showed that M(I + T) decreased the Kir2.1 current in mouse, with no change in rat. For anti-inflammatory activation states, there are even fewer publications. When TGF-β1 or TGF-β2 was used as a deactivating treatment on primary mouse microglia, there was no change in Kir current [25]. Here, we found that the Kir2.1 current was reduced by IL-4 in rat or IL-10 in mouse; changes that were consistent with the Kir2.1 transcript changes. For rat microglia, we previously reported a trend toward a decrease in current with IL-4 or IL-10 treatment but it did not reach statistical significance [18].

### Changes in Kv1.3 expression and current in activated rodent microglia

Kv1.2, Kv1.3, and Kv1.5 transcripts and protein have been detected in primary rat and mouse microglia [28, 29, 63–65]. Here, we detected all three channel transcripts in unstimulated rat and mouse microglia but Kv1.5 was very low. Some studies have suggested that elevated Kv1.3 indicates a pro-inflammatory state; e.g., increases in mRNA and channel expression in M(LPS) rat microglia [29, 96], and the present increase in Kv1.3 transcript expression only in M(I + T) mouse microglia. However, for rat microglia, we now show that Kv1.3 expression increased in both M(I + T) and M(IL-4) cells; thus, this channel gene is not a reliable marker of a pro-inflammatory state. Kv1.2 was striking in showing opposite responses to M(I + T): an increase in mouse but a decrease in rat microglia. The only other related studies on Kv transcript expression we found were on mouse cell lines. Kv1.2 mRNA expression increased in M(LPS) BV-2 cells [64], and Kv1.3 increased after TGF-β1 or TGF-β2 treatment in BV-2 and C8-B4 cells [25, 88].

There is considerable variability in the prevalence of Kv currents reported in primary rodent microglia. For unstimulated microglia, early reports showed an absence in rat [89, 97], presence in a small proportion (<10%) of rat and mouse cells [27, 95] or moderate prevalence (30%) in rat cells [93], while several later studies found a Kv current in most to all rat microglia [28, 29, 50, 90, 96, 98]. Most studies have not used selective blockers to isolate the Kv1.3 portion of the current. Here, we observed an AgTx-2-sensitive Kv1.3 current in all unstimulated rat microglia, which were predominantly unipolar, and this is consistent with our earlier studies on unstimulated bipolar or amoeboid rat microglia [26, 28, 50]. In contrast, despite the similar morphology and molecular profile of unstimulated mouse microglia, the Kv current prevalence was much lower (~56% of cells). A margatoxin (MgTx)-sensitive Kv current was seen in <10% of cultured mouse microglia [27] but that current was not proven to be Kv1.3 because MgTx can block other Kv1-family channels [99]. There is some evidence that the Kv prevalence changes with age in mouse but this has not been examined in rat. In mice, a MgTx- and AgTx-2-sensitive Kv current was seen in 20–60% of postnatal (P5–9) microglia in acutely isolated brain slices [100]. Little or no Kv current was detected in acutely isolated adult mouse brain slices [100–102] or microglia isolated from “control” mouse brain [103], but the prevalence increased to 29% in dystrophic microglia from aged mice [102]. A further complication is that the Kv prevalence and magnitude increased when mouse hippocampal slices were cultured ex vivo [101].

Some variability in the prevalence and amplitude of Kv currents is very likely due to differences in voltage protocols; e.g., using a depolarized holding potential, which is well known to inactivate Kv1.3 [26, 28, 93] and Kv1.5 channels [28, 104]. One study that did not detect Kv current in rat microglia used a holding potential of −20 mV [21]. Because Kv1.3 undergoes pronounced cumulative inactivation [28, 50–52], the current amplitude can be substantially underestimated if the interval between voltage pulses is too short. Most studies do not state the interpulse interval [21, 27, 89, 95]. Additional variability in Kv current might also reflect a different initial activation state but this has rarely been determined. For instance, both Kv1.3 and Kv1.5 currents have been identified in rat and mouse microglia [28, 65] but, in rat microglia from hippocampal tissue prints, Kv1.5 initially produced the current and was replaced by Kv1.3 as the cells became more proliferative [28].

Both Kv1.3 and Kv1.5 currents have been reported in ex vivo rat microglia [28] and in M(LPS) mouse microglia [65]. We previously provided the first evidence for a role for Kv1.3 in microglia, where it contributed to proliferation shortly after preparing ex vivo tissue prints from rat brain slices [28]. Changes in Kv current with activating stimuli suggest that it is induced or increased in pro-inflammatory states. LPS or IFN-γ increased Kv currents in rat [29, 52, 92, 93] and mouse microglia [27, 65, 95, 105]. Here, we found that the pro-inflammatory M(I + T) state increased the total Kv current and Kv1.3 component in both species. However, for rat microglia, the Kv1.3 increase was not specific to the pro-inflammatory state, as IL-4 also increased the current. Consistent with Kv1.3 mRNA expression, the pro-inflammatory response was more specific in mouse cells, where only I + T increased Kv and Kv1.3 currents. Kv currents were also prevalent in “activated” microglia in damage models but the specific activation state and channel type were not determined; for instance, in the denervated rat facial nucleus [21], and in the ischemic cortex [22]. MgTx- and AgTx-2-sensitive Kv1.3 currents were found in microglia within the hippocampus of adult mice after status epilepticus but the prevalence and cell activation state were not determined [23]. After LPS injection into the mouse brain, the PAP-1-sensitive Kv1.3 current in acutely isolated adult microglia was fivefold larger [103]. The present results show that additional Kv channel types were active in mouse microglia; i.e., less than half the current was blocked by AgTx-2 in unstimulated cells and slightly more than half in M(I + T) cells.

Little is known about Kv currents in anti-inflammatory states. Mouse microglia stimulated with TGF-β1 or TGF-β2 had increased Kv currents that were blocked by kaliotoxin, charybdotoxin, or MgTx [25, 88] but the cell activation state was not characterized. In both species, we found that the total Kv and isolated Kv1.3 currents were unchanged in M(IL-4) and M(IL-10) states.

### Contributions of Kir2.1 and Kv1.3 to microglial migration

For rat microglia, we previously reported that M(LPS) cells migrate less than unstimulated cells, while M(IL-4) and M(IL-10) cells migrate more [18–20, 30], and that blocking Kir2.1 channels reduced migration in unstimulated, M(IL-4) and M(IL-10) microglia [18]. We now report similar changes in migratory capacity with activation state in both species, and that, regardless of the activation state, blocking Kir2.1 inhibited migration while blocking Kv1.3 increased it. In the only directly relevant paper that we found, blocking Kv1.3 (and possibly other K^+^ currents) with MgTx in rat microglia reduced chemotaxis induced by monocyte chemoattractant protein 1 (CCL2) or ADP but, again, the activation state was not identified [106].

One potential role for Kv and Kir channels is to regulate the membrane potential and subsequent Ca^2+^ signaling, which is involved in cytoskeletal remodeling, adhesion and migration [107, 108]. For rat microglia, we previously showed that Ca^2+^ entry through Ca^2+^ release-activated Ca^2+^ (CRAC) channels is increased with hyperpolarization [109], and that blocking Kir2.1 channels with ML133 reduced CRAC-mediated Ca^2+^ entry and migration [18]. Similarly, blocking Kir2.1 in rat microglia with Ba^2+^ prolonged depolarization and reduced the amplitude of ATP-induced Ca^2+^ transients [110]. Blocking Kv1.3 in rat microglia disrupted membrane potential oscillations, showing a role in repolarization after depolarizing events [90]. This is apparently the first report of a role for Kv1.3 in microglia migration in the absence of a chemoattractant, and it was surprising that blocking Kv1.3 and Kir2.1 had opposite effects. Further evidence that the two channels do not always play the same functional roles is that Kir2.1 (but not Kv1.3) is involved in myelin phagocytosis and ROS production in activated rat microglia [17]. One possibility is that the channels have roles other than regulating K^+^ flux and membrane potential. For instance, cell migration requires integrins, which regulate adhesion to the extracellular matrix, and there is evidence that integrins and K^+^ channels can co-exist in signaling complexes [111]. A physical link between Kv1.3 and the β_1_ integrin moiety was reported in T lymphocytes and melanoma cells [112, 113] but it is not known whether a similar association occurs in microglia. Future studies will be needed to determine the mechanisms by which Kir2.1 and Kv1.3 channels regulate microglia migration.

### Limitations and future directions

1. Many molecules were analyzed using gene profiling to assess microglial activation responses. While mRNA levels are often well linked to protein levels, this is not always the case. Several interesting changes were further assessed at the protein and functional level. While it is important that channel proteins were assessed at the level of functional expression (ion currents), their role in only one cell function was examined (migration). In future, it would be valuable to assess roles of proteins in other microglial functions in the different activation states; e.g., relating ECM-degrading enzymes to invasion (as in [19]), relating phagocytosis receptors and NOX enzymes to phagocytosis and ROS production ([17]). 2. We assessed one time point (24 h for gene expression), which was chosen to facilitate comparisons with published gene analyses that include 24 h in vitro. For rat microglia, we knew that many genes respond at this time and, here, we found that some genes responded similarly in both species. However, it is possible that other responses, such as some non-responding genes in mouse cells reflect a different timing, and in future, one could examine the time course. 3. It is important to compare responses of both species to stimuli that are physiologically relevant to acute CNS damage in vivo. We used single cytokines or I + T but did not examine repolarization between M1 and M2 states, as we had previously done ([17]). The ability to compare with the literature is reduced because many studies have used LPS from gram-negative bacteria as the stimulus. 4. Although this study used primary cultured microglia (rather than cell lines), it was an in vitro study. We believe the results will at least aid in selecting and interpreting markers and molecules in animal models of CNS disease. Many rodent in vivo studies have presented evidence for M1 and M2 states of microglia (and macrophages) in both acute damage and chronic disease scenarios. For example, recent in vivo profiling shows gene expression and immunohistochemical evidence for M1 and M2a states in injured brain tissue [114–120]. Based on in vitro responses to cytokine stimulation, studies are increasingly using cytokines or their inhibitors in vivo; e.g., to promote the M2 phenotype [55, 121, 122]. Ultimately, it will be important to assess how rodent in vivo studies relate to human disease. 5. This study examined expression of numerous genes, verified changes of several at the protein level, and examined protein function for iNOS, Kv and Kir channels. However, in future, it would be valuable to examine the functionality of other proteins, such as the ROS-producing NOX enzymes.

### Broader implications

Studies of disease mechanisms rely on animal models, especially rodents. To fulfill the larger goal of translating such results to humans, it is crucial to understand whether rats and mice respond the same and, if not, which species is a more reliable model. There is considerable debate as to how closely mouse models resemble human responses in inflammatory diseases [123, 124]. While some in vivo results suggest that CNS inflammation differs between rats and mice, it is premature to ascribe the differences to microglial responses. For peripheral immune cells, rat and mouse responses are sometimes assumed to be comparable [125]. However, very few species comparisons have been published, and our findings illustrate important differences in molecular activation profiles between rat and mouse microglia. This might help explain why, after CNS injury/disease, some “hallmark” microglial activation state markers have been detected by immunohistochemistry in mouse but not in rat. While it is sometimes assumed that the antibodies do not work in rat (despite manufacturers’ claims), it might be that the species responses actually differ.

Microglial properties are increasingly being investigated in human tissue—often in surgical biopsies from epileptic patients—but the cell activation state is usually not determined and they cannot be assumed to be normal, resting cells. Further complications include the potential for strain differences in rodents [126], and genetic polymorphisms and epigenetic changes in humans [127]. Increasingly, in vivo studies include injection of a single stimulus (e.g., LPS, IFNγ, IL-4, IL-10, IL-13) in an attempt to skew the brain toward one activation state; however, measured brain responses reflect cell-cell interactions and numerous cell types. In vitro studies are the only way to stimulate just the microglia in order to elucidate similarities and differences in how different species respond. The information gained will be useful to help interpret results of previous and future in vivo studies. Many potential treatments identified in rodents have failed in human clinical trials. To narrow this translational gap, it is essential to investigate and acknowledge species similarities and differences in immune responses.

## Conclusions

The present study contributes considerable comparative data concerning primary rat and mouse microglia, and it highlights species similarities and differences in the inflammatory response following stimulation with pro- and anti-inflammatory cytokines. The search for molecular targets to control microglial activation and specific functions will also require a better understanding of species differences. For instance, Kv1.3 is considered a promising target in autoimmune diseases, such as multiple sclerosis, rheumatoid arthritis, type 1 diabetes, and psoriasis [128]. However, studies are only beginning to directly compare contributions of ion channels in different species. It is intriguing that, despite species differences in the outcome of microglial activation states on Kir2.1 and Kv1.3 expression and currents, there was no obvious species differences in the channel roles in migration. To determine whether specific microglial K^+^ channels can be targeted to modulate neuroinflammation, it will be crucial to undertake species comparisons of other microglia functions using selective inhibitors or cell-targeted channel depletion, and to extend the studies to models of acute CNS injury.

## Additional files

Additional file 1: Transcript expression of pro-inflammatory mediators. Rat and mouse microglia were unstimulated (CTL) or stimulated with IFN-γ and TNF-α (I + T), IL-4 or IL-10 for 24 h. mRNA counts for each gene were normalized to two housekeeping genes (described in Methods) and are shown as mean ± SEM (*n* = 4–6 individual cultures). *p < 0.05; **p < 0.01; ***p < 0.001; ****p < 0.0001 (PDF 386 kb)

Additional file 2: Transcript expression of anti-inflammatory mediators. Rat and mouse microglia were unstimulated (CTL) or stimulated with IFN-γ and TNF-α (I + T), IL-4 or IL-10 for 24 h. mRNA counts for each gene were normalized to two housekeeping genes (described in Methods) and are shown as mean ± SEM (*n* = 4–6 individual cultures). *p < 0.05; **p < 0.01; ***p < 0.001; ****p < 0.0001 (PDF 364 kb)

Additional file 3: Transcript expression of anti-inflammatory cytokines and their receptors. Rat and mouse microglia were unstimulated (CTL) or stimulated with IFN-γ and TNF-α (I + T), IL-4 or IL-10 for 24 h. mRNA counts for each gene were normalized to two housekeeping genes (described in Methods) and are shown as mean ± SEM (*n* = 4–6 individual cultures). *p < 0.05; **p < 0.01; ***p < 0.001; ****p < 0.0001 (PDF 380 kb)

Additional file 4: Transcript expression of microglia markers and immune modulators. Rat and mouse microglia were unstimulated (CTL) or stimulated with IFN-γ and TNF-α (I + T), IL-4 or IL-10 for 24 h. mRNA counts for each gene were normalized to two housekeeping genes (described in Methods) and are shown as mean ± SEM (*n* = 4–6 individual cultures). *p < 0.05; **p < 0.01; ***p < 0.001; ****p < 0.0001 (PDF 473 kb)

Additional file 5: Transcript expression of phagocytosis and purinergic receptors and NOX enzymes. Rat and mouse microglia were unstimulated (CTL) or stimulated with IFN-γ and TNF-α (I + T), IL-4 or IL-10 for 24 h. mRNA counts for each gene were normalized to two housekeeping genes (described in Methods) and are shown as mean ± SEM (*n* = 4–6 individual cultures). To facilitate species comparisons, the Y-axis was the same except as indicated by blue boxes. *p < 0.05; **p < 0.01; ***p < 0.001; ****p < 0.0001 (PDF 322 kb)

## Abbreviations

AgTx-2: Agitoxin-2
ARG1: Arginase 1
CCL: Chemokine (C–C motif) ligand
CD: Cluster of differentiation
Cm: Membrane capacitance
COX: Cyclooxygenase
CRAC: Calcium release-activated calcium channels
CT: Threshold cycle
CTL: Control
CXCL: Chemokine (C–X–C motif) ligand
DAPI: 4′,6-Diamidino-2-phenylindole
dNTP: Deoxynucleiotide tri-phosphate
EGTA: Ethylene glycol bis(2-aminoethyl ether)-N,N,N′N′-tetracetic acid
FBS: Fetal bovine serum
FIZZ1: Found in inflammatory zone 1
HEPES: 4-(2-Hydroxyethyl)-1-piperazineethanesulfonic acid
I: Current
Iba1: Ionized Ca2+ binding adapter molecule 1
ICE: Interleukin-1 converting enzyme
IFN-γ: Interferon gamma
IL: Interleukin
iNOS: Inducible nitric oxide synthase
Kir: Inward-rectifier potassium channel
Kv: Voltage-gated potassium channel
LPS: Lipopolysaccharide
M-CSF: Macrophage colony-stimulating factor
MEM: Minimum essential medium
MgTx: Margatoxin
ML133: N-(4-Methoxybenzyl)-1-(naphthalen-1-yl)methanamine
MRC1: Mannose receptor, C Type 1
NCF1: Neutrophil cytosolic factor 1
NO: Nitric oxide
NOX: Nicotinamide adenine dinucleotide phosphate-oxidase oxidase
OGD: Oxygen glucose deprivation
PAP-1: 5-(4-Phenoxybutoxy)psoralens
PBS: Phosphate-buffered saline
PPAR- γ: Peroxisome proliferator-activated receptor gamma
PYK2: Protein Tyrosine kinase 2 beta
ROS: Reactive oxygen species
SHP-1: Src homology region 2 domain-containing phosphatase-1
SOCS: Suppressor of cytokine signaling
SR-A: Scavenger receptor-A
TGF-β: Transforming growth factor beta
TLR: Toll-like receptor
TNF-α: Tumor necrosis factor alpha
TREM2: Triggering receptor expressed on myeloid cells 2
TSPO: Translocator protein
Vm: Membrane potential
