# Supplementary material for: Responses of rat and mouse primary microglia to pro- and anti-inflammatory stimuli: molecular profiles, K+ channels and migration
Source: J Neuroinflammation. 2017 Aug 22;14:166. doi: 10.1186/s12974-017-0941-3 (PMC5567442; doi:10.1186/s12974-017-0941-3)
Supplement: Supplementary file 4 — Transcript expression of microglia markers and immune modulators. Rat and mouse microglia were unstimulated (CTL) or stimulated with IFN-γ and TNF-α (I + T), IL-4 or IL-10 for 24 h. mRNA counts for each gene were normalized to two housekeeping genes (described in Methods) and are shown as mean ± SEM (n = 4–6 individual cultures). *p < 0.05; **p < 0.01; ***p < 0.001; ****p < 0.0001 (PDF 473 kb) [file 12974_2017_941_MOESM4_ESM.pdf]

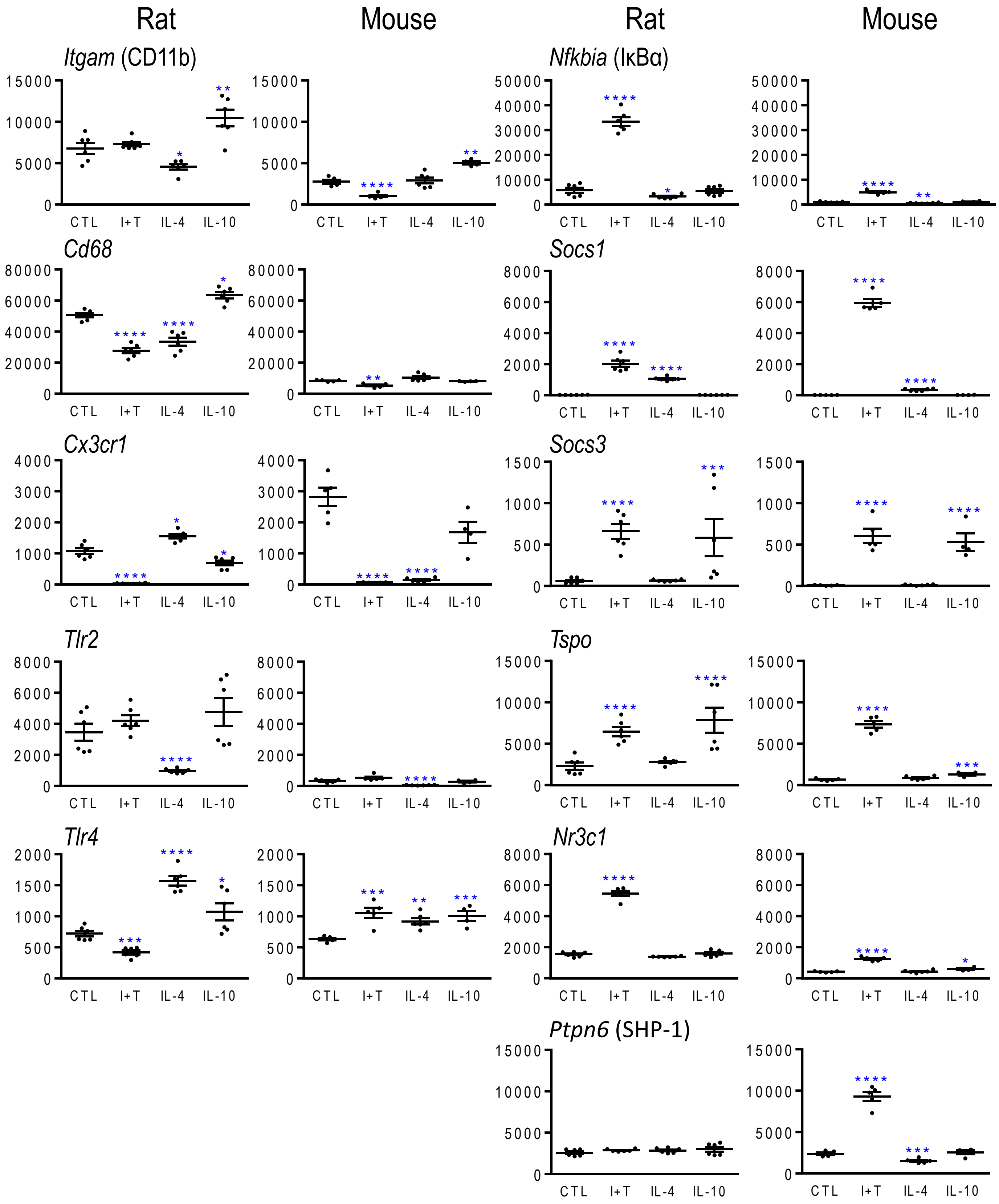

Additional File 4. Transcript expression of microglia markers and immune modulators. Rat and mouse microglia were unstimulated (CTL) or stimulated with IFN-γ and TNF-α (I+T), IL-4 or IL-10 for 24 h. mRNA counts for each gene were normalized to two housekeeping genes (described in Methods) and are shown as mean ± SEM (n=4–6 individual cultures). *p<0.05; **p<0.01; ***p<0.001; ****p<0.0001
